# Fabrication of Ag@Co-Al Layered Double Hydroxides Reinforced poly(o-phenylenediamine) Nanohybrid for Efficient Electrochemical Detection of 4-Nitrophenol, 2,4-Dinitrophenol and Uric acid at Nano Molar Level

**DOI:** 10.1038/s41598-019-49595-y

**Published:** 2019-09-13

**Authors:** T. Dhanasekaran, R. Manigandan, A. Padmanaban, R. Suresh, K. Giribabu, V. Narayanan

**Affiliations:** 1 Department of Inorganic Chemistry, University of Madras, Chennai, India; 2 National Centre for Sustainable Coastal Management, Anna University Campus, Chennai, India; 3 School of Electronic Science and Engineering, University of Electronic Science and Technology of China, Chengdu, China; 4 Department of Analytical and Inorganic Chemistry, University of Concepcion, Concepcion, Chile; 5 Electrodics and Electrocatalysis Division, CSIR-CECRI, Karaikudi, India

**Keywords:** Biocatalysis, Electrocatalysis, Bioinspired materials

## Abstract

In this paper, Co-Al layered double hydroxides (LDHs), Co-Al LDHs/poly(o-phenylenediamine) (PoPD) and Ag nanoparticles decorated Co-Al LDHs/PoPD (Ag@Co-Al LDH/PoPD) samples were prepared. The as-prepared samples were characterized by XRD, Raman, XPS, FT-IR, DRS-UV-Vis, PL and TGA techniques. The salient features of morphology and size of the samples were determined using FESEM, and HR-TEM. Then, the samples were coated on glassy carbon electrode (GCE) and employed for sensing of 4-nitrophenol (4-NP), 2,4-dinitrophenol (2,4-DNP)) and uric acid (UA). It was found that Ag@Co-Al LDH/PoPD/GCE showed superior electrochemical sensing behaviour than other modified electrodes. It exhibits the detection limit (DL) of 63 nM, 50 nM and 0.28 µM for 4-NP, 2,4-DNP and UA respectively.

## Introduction

The detection of explosives has received great attention in the perspective of environmental application and national security. The secondary explosives are most prevalent at military sites compared to primary explosives^1^. The examples of secondary explosives are dinitrotoluene (DNT), trinitrotoluene (TNT), 4-nitrophenol (4-NP), 2,4-dinitrophenol (DNP), 2,4,6-trinitrophenol (TNP), and hexahydro-1,3,5 trinitroazine (RDX)^2^. These pollutants are having nitro (NO_2_) group that is highly hazardous to the environment due to its interaction with the biota. Hence, the sensitive and rapid determination of these pollutants is significantly important. In this regard, many research groups were trying to develop new detection methods with high reliability, sensitivity and selectivity^3^. So far, several techniques such as gas chromatography-mass spectroscopy (GC-MS)^4^, surface enhanced Raman spectroscopy (SERS)^5^, liquid chromatography-mass spectroscopy (LC-MS)^6^, fluorescence^7^, proton transfer reaction-mass spectrometry^8^ and ion mobility spectrometry^9^ have been developed to detect the nitro explosives. Among these techniques, the electrochemical analysis^10^ possesses several advantages such as low cost, easy operation, high sensitivity, and suitability for fabrication of portable devices.

An aromatic nitro compounds such as 4-NP, and 2,4-DNP are not only used in explosives, but also in pesticides^11^ and they discharged into environment. Thus, detection of these pollutants is of great importance for environmental pollution monitoring. In earlier, an amperometric determination of 4-NP was developed by Kubota’s group using a modified GCE^12^. Recently, Mehdinia *et al*. established a method to detect 4-NP using a molecularly imprinted polymer (MIP)^13^. Likewise, there are numerous reports available for sensing of 4-NP and 2,4-DNP. Still there is an urgent need to develop a protocol to quantify 4-NP and DNP at trace level.

On the other hand, UA is an important biomarker produced by purine metabolism in our body. In an accordance of the health care for humans, the excretion and production of UA is dynamic balance under normal circumstances^14^. The tricky levels of UA in the body could affect the hyperuricemia, gout, and Lesch–Nyhan syndrome^15^. Hence, developing reliable and sensitive methods for UA detection is necessary.

Layered double hydroxides (LDHs) play an important role as a host matrix in the several fields such as electrochemical sensors^16^, fuel cells^17^, capacitors^18^ and catalysis^19^. LDHs are well-known materials because of their low cost, high chemical stability, catalytic activity, and biocompatibility and intercalation properties^20^. The conducting polymers also have significant interest in various applications such as electronic, electrical and optical devices^21^. Among the conducting polymers, Poly(o-Phenylenediamine) (PoPD) is one of the most thoroughly studied substance because of its ease of synthesis, stability, high conductivity and good dispersibility^22^. In recent years, metal nanoparticles (MNPs) have been emerged as an auspicious material. They exhibit inspiring potential in electrochemical sensor due to their excellent electrical conductivity. The Ag nanoparticles display good catalytic ability towards oxidation-reduction reaction of nitro compounds and biomolecules owing to their effective mass transport ability and high surface area^23,24^.

**Figure 1:**
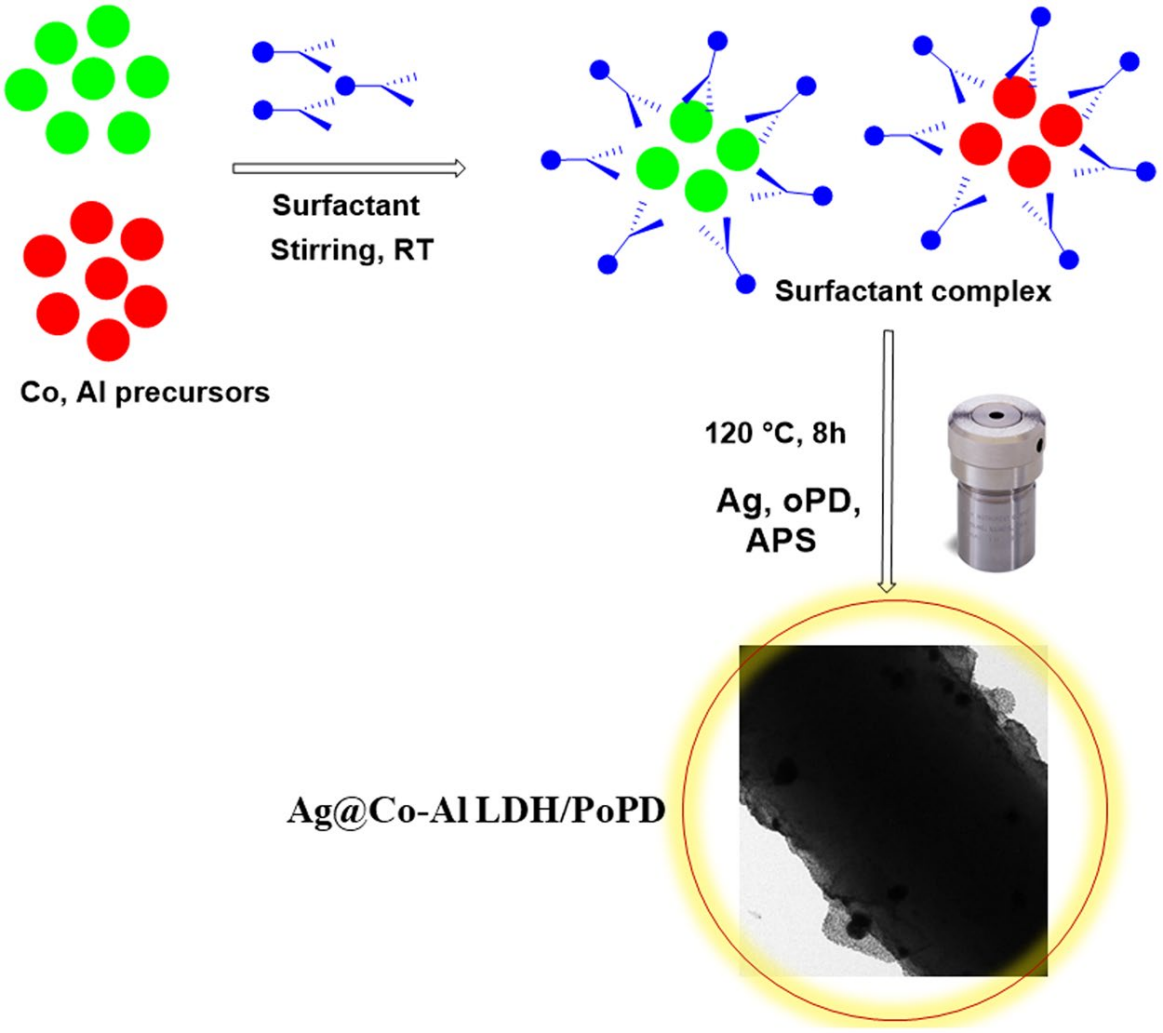
Schematic illustration for the synthesis of Ag@Co-Al/PoPD nanohybrids.

**Figure 2:**
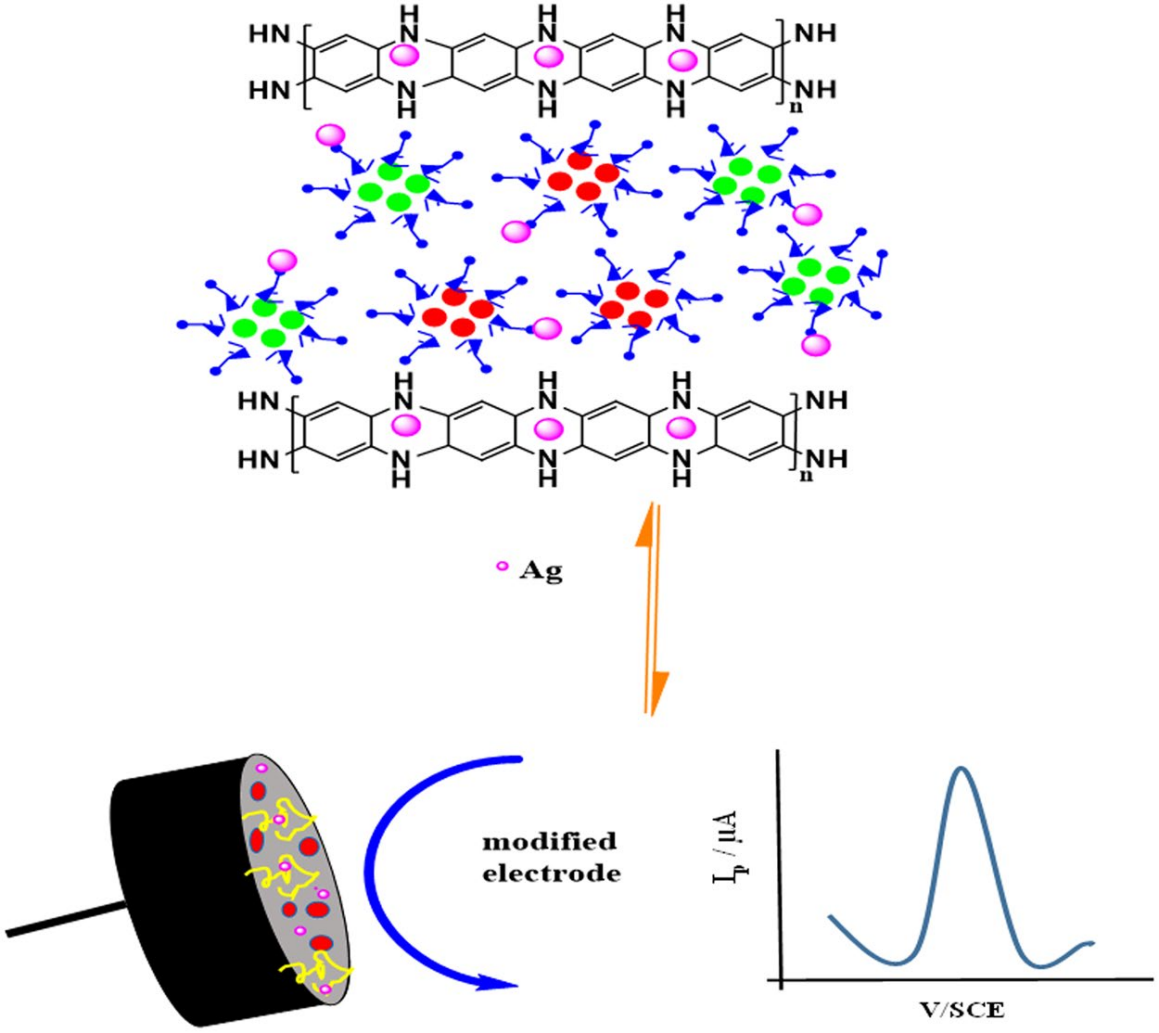
Graphical illustration of Ag@Co-Al/PoPD nanohybrid modified GCE.

Based on the above information, we motivated to fabricate Ag@Co-Al LDH/PoPD nanohybrids modified GCE. In this paper, we reported a simple and cost effective method to synthesize Ag@Co-Al LDH/PoPD (Figs 1 and 2). The nanohybrid modified GCE detects the 4-NP, 2,4-DNP and UA with highly sensitivity.

## Materials and Characterization Methods

### Reagents

Co(NO_3_)_3_.6H_2_O, Al(NO_3_)_3_·9H_2_O, Uric acid and Ag(NO_3_) were purchased from Sigma-Aldrich. 4-Nitrophenol (4-NP), 2,4-Dinitrophenol (2,4-DNP), o-phenylenediamine (o-PD), urea and NaOH were purchased from SRL company. Sodium phosphate monobasic anhydrous, sodium phosphate dibasic dihydrate, sodium acetate and acetic acid (analytical reagent grade) were bought from Sisco chemical, India. The solvents, ethanol, methanol and acetone were purchased from Merck and used without further purification. MilliQ water was used to prepare the all the solutions.

### Electrochemical experiment

The prepared 1 mM stock solutions (using MilliQ water) of 4-NP, 2,4-DNP and UA were stored at 4 °C. The pH from 3–9 were adjusted using sodium hydroxide and Sulphuric acid. All the chemicals (analytical grade) were used as received. The acetate buffer of pH-5 was used as background electrolyte for detection of 4-NP and 2,4-DNP but in the case of UA using pH-7 (phosphate buffer) was optimized and the pH ranged from 3–9.

### Fabrication of Ag@Co-Al LDH/PoPD nanohybrid modified glassy carbon electrode

The GCE’s surface was polished by using 1.0 and 0.3 µm alumina slurry. The dispersion of Ag@PoPD/LDH in ethanol obtained by using ultra-sonication for 15 min and then a 5 μL of dispersion was drop casted on the surface of polished GCE and dried at room temperature. Finally, the modified GCE used for further electrochemical analysis.

## Results and Discussions

**Figure 3:**
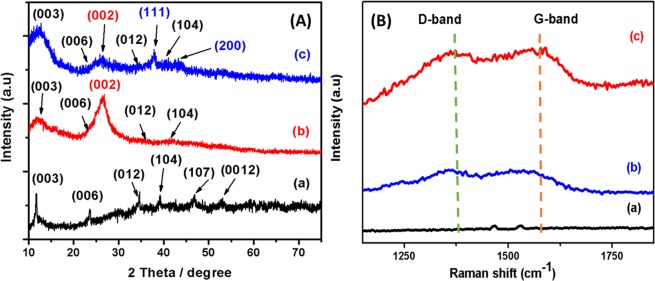
XRD pattern (**A**) and Raman spectra (**B**) of (a) pure Co-Al LDHs, (b) PoPD/LDHs and (c) Ag@Co-Al/PoPD.

The crystal structure and phase purity of the samples were determined from X-ray diffraction analysis. In Fig. 3A **(pattern a)**, the diffraction peaks of pure Co-Al LDH are well matched with JCPDS file no. 10-2794. It shows that the peaks at 11.75°, 23.62°, 34.71°, 39.36°, 46.97°, 53.24°, 60.38°, and 61.75° are corresponding to the reflection from (003), (006), (012), (104), (107), (0012), (0111) and (110) planes of the rhombohedral structured Co_6_Al_2_(OH)_16_·4H_2_O respectively. The formation of rhombohedral phase may be due to the coordination of aluminium with the cobalt ions, as reported in literature^25^. The basal plane (003) indicates the well-organized 2D layer stacking of the prepared LDHs. There are no peaks due to oxides of cobalt and aluminium in XRD patterns of samples.

The XRD pattern of Co-Al LDH/PoPD hybrid nanostructures is shown in Fig. 3A, pattern b. It shows characteristic broad peak at 26.25° (JCPDS no: 101-1061), due to periodicity parallel chain of PoPD^26^. It denotes amorphous nature of PoPD. In addition, characteristic peaks of Co-Al LDHs are not clearly observed. This is because of existence of amorphous PoPD particles. Figure 3A (pattern c) predominantly shows the diffraction peaks of Ag (cubic) in Ag@Co-Al/PoPD (JCPDS file no: 901-3047). The diffraction peaks at 37.93° and 44.08° are due to crystal planes of (111) and (200) respectively^27^. The suppression of broad diffraction of peaks of PoPD might be due to dominance of more intense peaks of silver. The average crystalline size was calculated using Scherrer equation^28^,

$$D=\frac{K\lambda }{\beta \,cos\theta }$$

where, λ is the wavelength, K is a shape factor, θ is the diffraction angle and β is the full width of half-maximum diffraction peak. The calculated average crystallite size is ~8 nm for Ag@Co-Al LDH/PoPD.

Raman spectroscopy is a powerful non-destructive technique to examine the imperfection and disordered crystal structures. The Raman spectrum of Co-Al LDHs shows the peaks at 482, 554, 1053, 1469 and 1536 cm^−1^ (Fig. SI). The sharp peaks at 482 and 554 cm^−1^ are due to stretching vibrations of metal-oxygen (M–O) bonds. The peak, 1053 cm^−1^ is due to stretching vibration of (CO_3_^2−^), which is commonly observed for CO_3_^2−^ intercalated LDH material^29^. The two broad peaks at 1362 and 1559 cm^−1^ are corresponding to the D- and G-bands, respectively. The G-band (E_2g_) assigned to stretching vibrations in the basal-plane (sp^2^ domains) of PoPD polymer^30^. Further, the D-bands are usually attributed to the disorders and imperfections in the carbon crystallites. However, the Raman bands due to LDH are not observed in Ag@Co-Al LDH/PoPD. The Raman spectrum of Ag@Co-Al/PoPD (Fig. 3B, spectrum c) shows two peaks at 1355 and 1530 cm^−1^ that indicate the presence of PoPD. The intensity of two broad peaks due to D- and G-band is less. This is because of addition of Ag nanoparticles, which might be suppressed M-O peaks intensity^31^.

**Figure 4:**
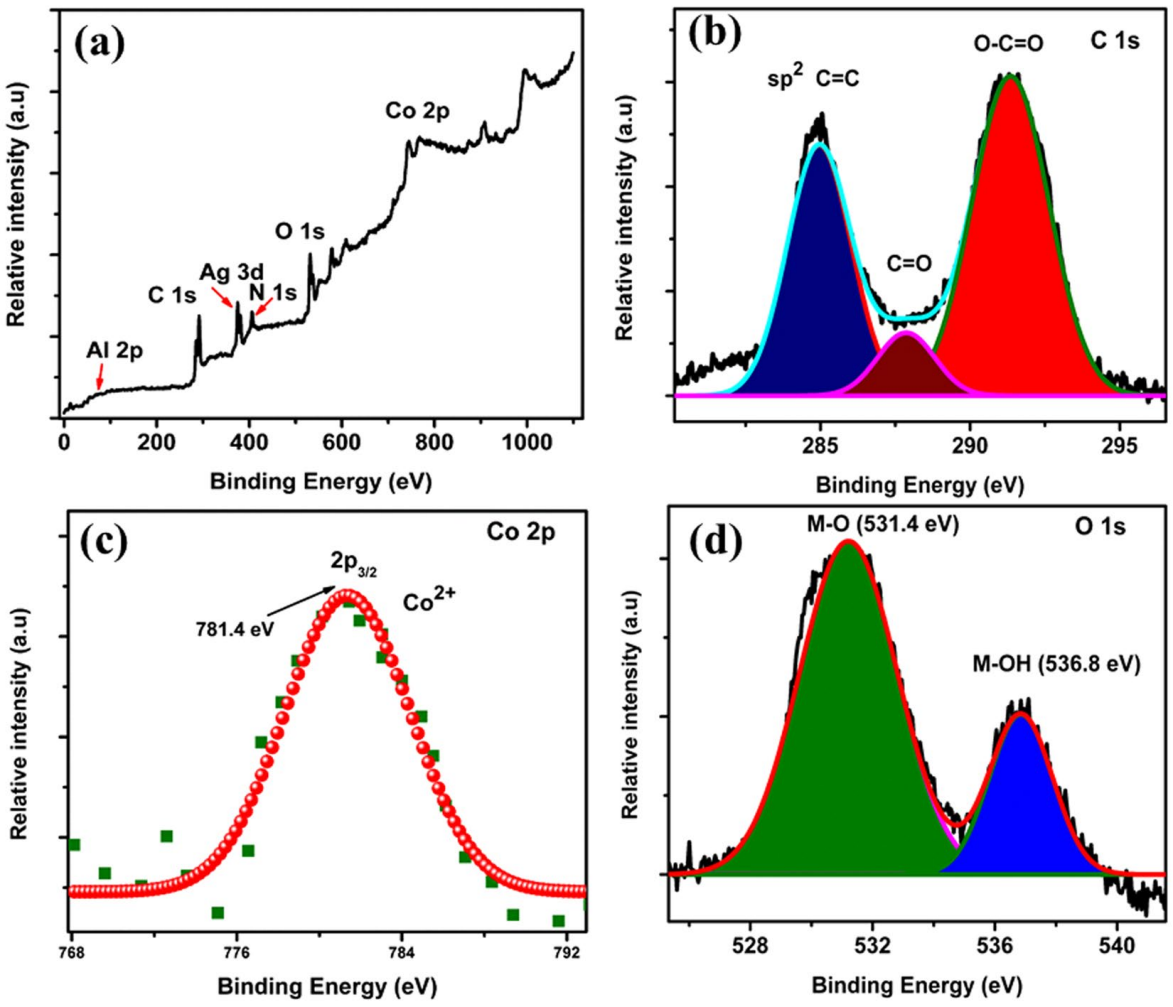
The XPS survey spectrum (**a**) and core-level spectrum of C 1 s (**b**), Co 2p (**c**) and O 1 s (**d**).

The elemental composition and their oxidation states of Ag@Co-Al LDH/PoPD were analysed by XPS. The survey spectrum and core-level spectrum of C 1 s, Co 2p and O 1 s are given in Fig. 4(a–d). Obviously, the Ag@PoPD/Co-Al LDH showed predominant peaks due to Ag 3d, Al 2p, N 1s  spectrum peaks (SI. Fig. 2a–c). The core level C 1 s spectrum (Fig. 4b) was deconvoluted into three peaks. These peaks are correspond to aromatic linked carbon (C=C, 284.9 eV), the carbonyl carbon (C=O, 287.8 eV) and the carboxylate carbon (O-C=O, 291.35 eV). Generally, the core level spectrum of Co 2p exhibits the spin-orbit split doublet. However, in our case, only a peak of Co 2p 3/2 can be observed at 781.4 eV (Fig. 4c**)**^32^. This result is in accordance with XRD and SAED analysis. The deconvoluted core level spectrum of O 1 s shows (Fig. 4d**)** two peaks which are attributed to M-O bonds (531.4 eV), and surface hydroxyl group (536.8 eV).

**Figure 5:**
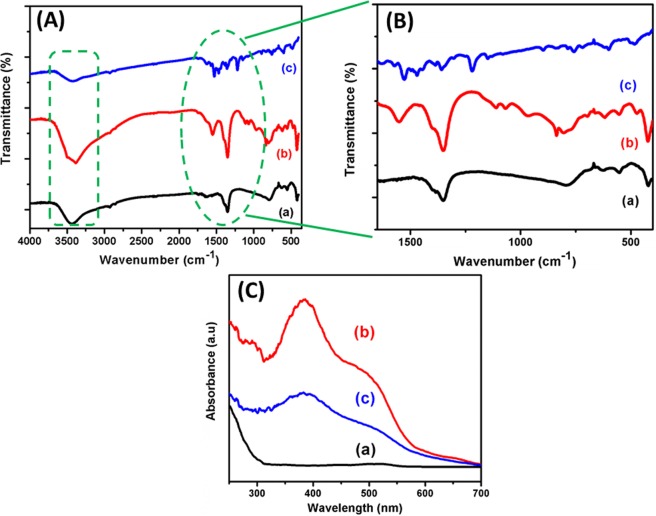
(**A,B**) FT-IR and (**C**) DRS-UV-vis spectra of (a) pure Co-Al LDH, (b) Co-Al/PoPD and (c) Ag@Co-Al/PoPD samples.

Figure 5A,B(a–c) displays the FT-IR spectra of Co-Al LDH, Co-Al LDH/PoPD and Ag@Co-Al LDH/PoPD nanohybrids. Figure 5A (spectrum a) shows the peaks at 3440, 1633, 1351, 779, 554 and 420 cm^−1^. The peak in range of 3500–3250 cm^−1^ is related to O-H stretching vibrations adsorbed water molecule, whereas bending vibration of O-H is observed at 1633 cm^−1^. The peak, 1351 cm^−1^ is assigned to stretching of CO_3_^2−^ group with a double bond resonance character respectively. It should be mention that this peak is observed in all the samples^33^. The peaks at 779, 554 and 420 cm^−1^ are attributed to Co-O-Al, Co-O and Al-O vibrations respectively. Figure 5A (spectrum b) represents the broad peak at 3385 cm^−1^ that is due to N-H stretching vibration. The peaks at 1564 and 1351 cm^−1^ are attributed to C=N and C=C stretching mode of protonated quinoid (Q) and benzenoid (B) rings, respectively^34^. The peaks at 1107, 1061, and 969 cm^−1^ are assigned to CO_3_^2−^, C-N and C-H vibrations respectively. The weak peak observed at around 850–745 cm^−1^ is due to C-O-M bond and the metal-oxygen bond appeared at the lower frequency range (900–400 cm^−1^). Moreover, Fig. 5A (spectrum c) exhibits broad peaks at 3400–3500 cm^−1^ (ʋ (O-H)) and 1630 cm^−1^ (δ(H_2_O)) attributed to the intercalated water molecules. The observed peaks at 1530, 1465, 1651and 1224 cm^−1^ correspond to the PoPD polymer (Fig. 5B). The C-O-Co stretching vibration is observed at 763 cm^−1^ and the peak at 603 and 481 cm^−1^ may be due to Al-O and Ag-O bonds^35^.

The absorption spectra of pure Co-Al LDH, Co-Al LDH/PoPD and Ag@Co-Al LDH/PoPD nanohybrid are shown in (Fig. 5C, spectra a–c). The Co-Al LDH shows a weak peak at 515 nm, due to n-π* transition (Fig. 5C, spectrum a). The broadening of absorption range may arise due to (i) electrostatic interaction (ii) H-bonding formation between guest-host molecules and (iii) van der Waals force of attraction. However, in the case of Co-Al LDH/PoPD, three main peaks are observed at 290, 385 and 490 nm (Fig. 5C, spectrum b). The peaks at 290 and 385 nm are due to π-π* electronic transition of conjugated C=C double bond^36^. The notable blue shift is due to the quantum confinement effect that occurred during the PoPD growth.

The sharp optical absorption edges and well-defined excitonic features indicated that the synthesized Co-Al LDH particles have relatively narrow size distribution. The peak at 490 nm of the Co-Al/PoPD is slightly shifted to lower wavelength with respect to Co-Al LDH, indicating the formation of nanohybrids. Whereas, the absorption peak at 515 nm of Co-Al LDH is blue shifted to 490 nm, this shift was attributed to the strong coupling effect between Co-Al LDH and PoPD. The UV visible absorption spectrum of Ag@Co-Al LDH/PoPD is shown in Fig. 5C, spectrum (c). It shows two main peaks at 386 and 496 nm. The broad peak at 386 nm and the weak peak at 419 nm is due to π-π* transition of C=C and surface plasmon resonance of Ag respectively^37^. The weak absorption peak appears at 496 nm is due to n-π* with respect to the LDH.

### Morphological features

**Figure 6:**
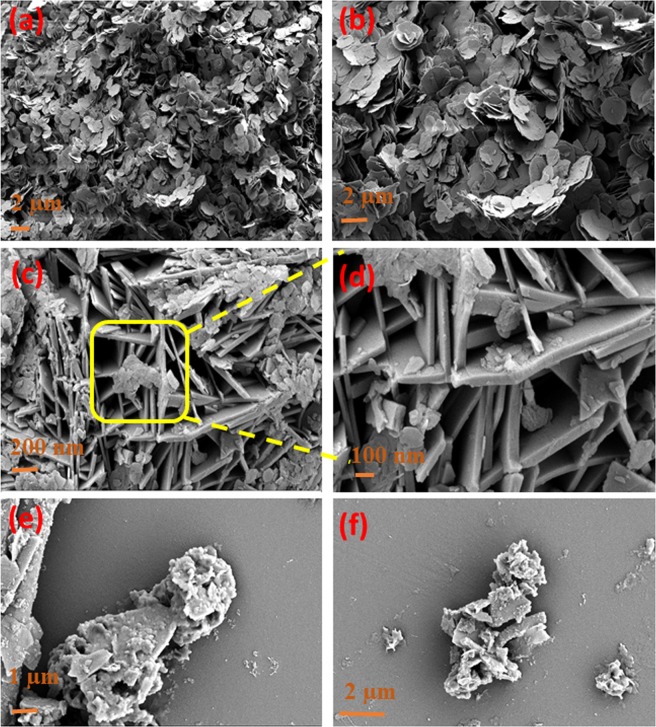
FESEM images of (**a,b**) pure Co-Al LDH, (**c,d**) Co-Al/PoPD and (**e,f**) Ag@ Co-Al/PoPD nanohybrids.

Figure 6(a,b) reveals that pure Co-Al LDH powder was composed of flakes like particles. Despite of the intercalated materials, high surface area with less aggregate has achieved for Co-Al LDH/PoPD sample (Fig. 6c,d). The high and low magnified FESEM images of Co-Al LDH/PoPD display that Co-Al LDH flakes are spread on the PoPD matrix. This is due to the interaction of metal centres of LDH with electron rich conjugated double bonds in PoPD. Figure 6(e,f) shows the FESEM images of Ag@Co-Al LDH/PoPD sample. The images show that the Ag nanoparticles are decorated on the Co-Al /PoPD nanohybrids, because of the surface interaction between the Ag and PoPD. The even distribution of Ag on Co-Al LDH/PoPD was further confirmed by elemental mapping analysis (Fig. S4). This result is well accordance with our XRD analysis. Furthermore (SI Fig. 5a,c,e) EDS spectrum and (b, d, f) FESEM images were shown in SI Fig. S5.

**Figure 7:**
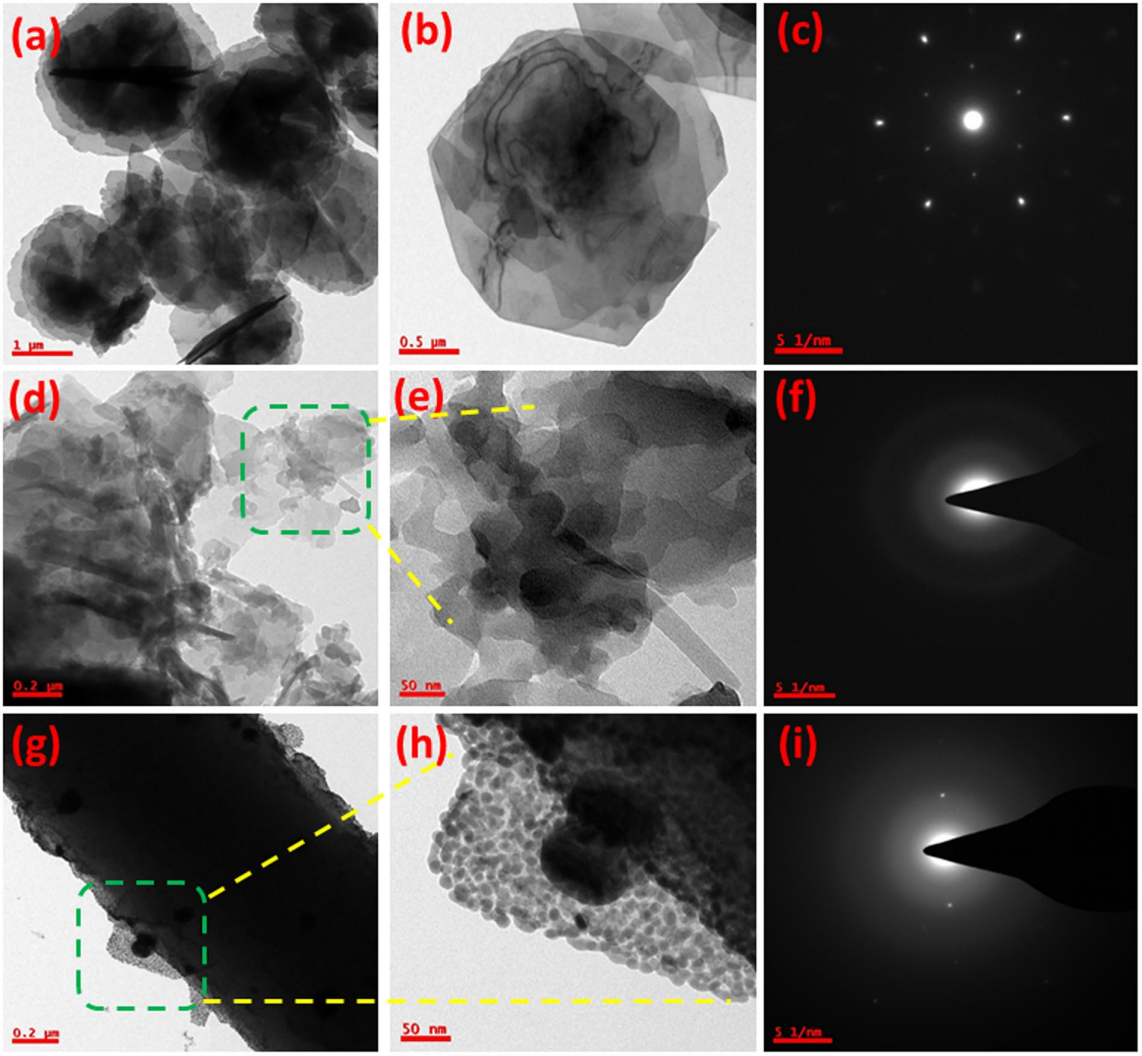
Low and high magnification HR-TEM images of (**a,b**) pure Co-Al LDH, (**d,e**) Co-Al/PoPD and (**g,h**) Ag@Co-Al/PoPD nanohybrids. The images, (**c**), (**f**) and (**i**) are SAED pattern of pure Co-Al LDH, Co-Al/PoPD and Ag@Co-Al/PoPD nanohybrids respectively.

The surface morphology of pure Co-Al LDH, Co-Al/PoPD and Ag@ Co-Al/PoPD were analysed using HRTEM images. The low and high-magnified HRTEM images of pure Co-Al LDH are shown in Fig. 7(a,b). The Co-Al LDH adopts hexagonal flake like morphology, which clearly indicates the formation of layered structure. The interaction between Co-Al hydroxides may be due to electrostatic interaction happen during growth of LDH as reported by Duan *et al*.^38^. From the Fig. 7d,e, we can observe that the flakes and rod-like particles with rough surface of the Co-Al LDH layer are attached on the surface of the PoPD nanoparticles. The deposition of Co-Al LDH on PoPD can be explain by the following manner: When the o-PD monomer added to the LDH dispersion containing HCl, the o-PD monomer gets protonated and becomes positively charged, which is adsorbed on the negatively charged LDH nanoparticles due to the strong electrostatic force of attraction. After adding, ammonium persulfate as an oxidant, the oxidative polymerization reaction takes place completely and thus obtained Co-Al LDH/PoPD nanohybrids. Figure 7(g,h) shows HR-TEM images of Ag@Co-Al LDH/PoPD surface morphology at high and low magnifications. The Ag nanoparticles with average diameter of <10 nm are homogeneously distributed on the surface of Co-Al LDH/PoPD nanostructures (Fig. S6a,b). The SAED pattern of Co-Al LDH, Co-Al/PoPD and Ag@Co-Al /PoPD were shown in Fig. 7(c,f,i). From SI Fig. 7(a–f), shown the lower magnification images and the calculated d-spacing values using fringes pattern, it is well matched with XRD analyses.

### Electrochemical sensing properties

#### Electrochemical sensing of 4-Nitrophenol

**Figure 8:**
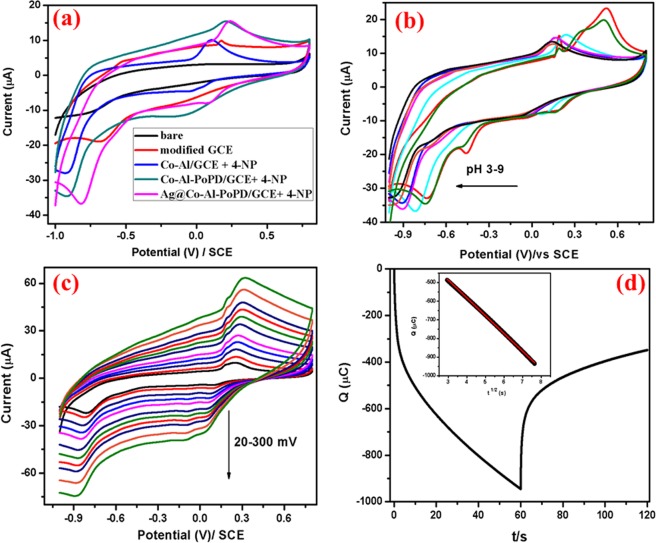
(**a**) Cyclic voltammograms of bare GCE (Black), Ag@Co-Al/PoPD/GCE (Red) in absence of 4-NP, Co-Al/GCE (Blue), Co-Al/PoPD/GCE (Green) and Ag@Co-Al/PoPD/GCE (Pink) in the presence of 1 × 10^−3^ M 4-NP (pH 5) at the scan rate of 50 mVs^−1^. (**b**) Effect of pH (pH 3-9) on 4-NP reduction at Ag@Co-Al/PoPD/GCE, (**c**) effect of scan rate (20–300 mVs^−1^) on 4-NP reduction at Ag@Co-Al/PoPD/GCE, and (**d**) Chronocoulometric curve of Ag@Co-Al/PoPD/GCE in presence of 4-NP (inset fig: t^1/2^ vs Q).

Electrochemical sensing of 4-NP: Fig. 8a displays the cyclic voltammograms of bare and modified electrodes in absence and presence of 1 × 10^−3^ M 4-NP. The CV of Ag@Co-Al LDH/PoPD/GCE in absence of 4-NP, shows single anodic peak at 0.18 V and no cathodic peaks were observed. In the presence of 4-NP, a pair of cathodic peaks and one anodic peak were observed. However, Co-Al LDH/GCE, Co-Al LDH/PoPD/GCE and Ag@Co-Al LDH/PoPD modified GCE show shift in cathodic peak potential for 4-NP with higher peak current and low potential than the bare GCE. The obtained reduction peak potential of 4-NP at modified GCE are as follows: Co-Al LDH [−0.04, 0.92 V], <Co-Al/PoPD [−0.14, 0.92 V] < Ag@Co-Al/PoPD [−0.08, 0.81 V]. All the corresponding data were tabulated in SI Table 1. From this, it is clear that the Ag@Co-Al LDH/PoPD/GCE has good electrochemical sensing behaviour towards the reduction of 4-NP. The reasons for enhanced sensing behaviour are due to the highly accessible active sites of the modifying layer due to nanosized dimensions of the samples as evident from HR-TEM images and excellent redox property of PoPD. Electrocatalytic sensing mechanism of 4-NP at Ag@Co-Al LDH/PoPD nanohybrids can be stated as follows: Based on the above discussion, the electrochemical redox reaction using Ag@Co-Al LDH/PoPD in presence of 4-NP can be expressed by following stages.

Step-I: The mass transport of 4-NP from bulk solution to the electrode surface.

Step-II: The adsorption of 4-NP on Ag@Co-Al LDH/PoPD/GCE through hydrogen bonding or electrostatic interactions and π-π interaction.

Step-III: The electron transfer reaction takes place.

Step-IV: Mass transport of product from the Ag@Co-Al LDH/PoPD/GCE surface into the bulk solution.

Based on the previous reports^39^, the redox pathway of 4-NP, which involves 6 electron process^40^, at Ag@Co-Al LDH/PoPD/GCE could be explain as follows:. In aromatic compounds (4-NP), it is known that nitro group is a well withdrawing and good leaving group, favouring electrophilic substitution reaction by the OH group present in the 4-NP at para position. The product (I) is observed at +0.23 V by the simultaneous substitution of the hydroxyl radical followed by oxidation, which is equivalent to that of the quinone and on reduction cycle, the product (II) is formed at −0.08 V and −0.81 V which is equivalent to that of hydroquinone. It indicates the further reduce the nitro groups into amino group.

**Figure 9:**
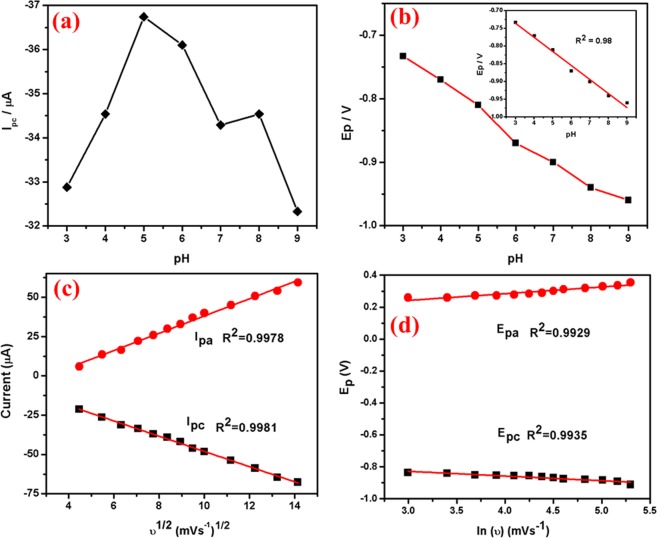
(**a**) The plot of pH versus Ip and (**b**) pH versus Ep, (**c**) square root of scan rate (ʋ^1/2^) versus Ip and (**d**) ln(ʋ) vs Ep for Ag@Co-Al/PoPD/GCE in 1 × 10^−3^ M 4-NP (pH = 5).

pH study: Fig. 9b shows the influence of pH (3–9) on the electrochemical response of Ag@Co-Al LDH/PoPD/GCE in 1 × 10^−3^ M 4-NP. The reduction peak potential of 4-NP increases gradually with increasing pH from 3.0 to 9.0, further increase in pH, diminish the reduction peak current. At pH = 5, the reduction peak current of 4-NP is greater when compared to that in other pH values. Hence, pH = 5 was chosen for the further studies. The reason for the higher reduction peak current may be attributed to the electrostatic attraction between the 4-NP and Ag@Co-Al LDH/PoPD. Figure 9(a,b) shows the plot of pH vs I_p_ and pH vs E_p_ plots for Ag@Co-Al LDH/PoPD).

Scan rate effect: Electrochemical reduction of 4-NP at different scan rates was examined to know about the kinetics of the electrode reaction. The CV’s of the 4-NP at Ag@Co-Al LDH/PoPD/GCE in (pH = 5) 1 × 10^−3^ M 4-NP at different scan rates (20–300 mV) is shown in Fig. 8c. Figure 9c shows the plots of anodic or cathodic peak current of υ^1/2^ vs. I_pa_ (µA) for Ag@Co-Al/PoPD/GCE. The slope value was found to be greater than 0.5 which denotes that redox process of 4-NP is adsorption controlled process. The linear regression equation of the plot is given as,

$${{\rm{I}}}_{{\rm{pc}}}=-\,0.678\,\log ({\rm{\upsilon }})+0.510\,({{\rm{R}}}^{2}=0.9981)$$

The relationship between ln vs. E_pc_ (V) is shown in Fig. 9d. The obtained linear regression equations is,

$${{\rm{E}}}_{{\rm{pc}}}=-0.040\,\log ({\rm{\upsilon }})-0.2410\,({{\rm{R}}}^{2}=0.9935)$$

According to the Laviron equation, the totally reversible electrode process of 4-NP, and the relationship between the potential (E_pc_) and scan rate (υ) could be expressed by the following equation^41^.

$$Epa={E}^{o}-\frac{RT}{(1-\alpha )\,nF}\,ln\,\frac{RTks}{(1-\alpha )\,nF}+\frac{RT}{(1-\alpha )\,nF}ln{\rm{\upsilon }}$$

The electrode process purely depends on the modifying layer (i.e., Ag@Co-Al LDH/PoPD/GCE). The overall redox reaction of 4-NP, the number of electrons involved and obtained by following equation

$$Epa=E-[\frac{2.303\,mRT}{nF}]pH$$

The number of electrons involved in this redox process is calculated as 6.

Chronocoulometry: Diffusion coefficient (D) value identified from chronocoulometry for Ag@Co-Al/PoPD nanohybrids is shown in Fig. 8d. The diffusion coefficient value was determined using the Cottrell equation^42^,

$$Q=\frac{2nFAc{D}^{\frac{1}{2}}{t}^{\frac{1}{2}}}{{\pi }^{\frac{1}{2}}}+{Q}_{ads}$$

Where, n = number of electrons in the reaction, Q = charge, A = electrode surface area, F = Faraday’s constant, D = diffusion coefficient and C_o_ = concentration of the system. The inset in Fig. 8d is the plot of square root of time (t^1/2^) against charge (Q) which showed a linear relationship. A = 0.07 cm^2^ (geometric area), n = 6 and c = 0.001 M, the value of the diffusion coefficient was calculated for Ag@Co-Al/PoPD/GCE nanohybrids, as D = 6.21 × 10^−12^ cm^2^ s^−1^.

**Figure 10:**
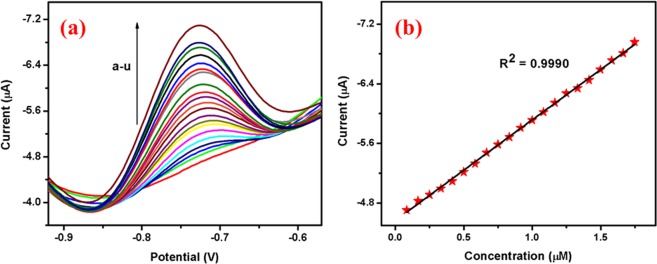
(**a**) Differential pulse voltammograms different concentraions of (from a to u: 5 µM - 105 µM) 4-NP at Ag@Co-Al/PoPD/GCE and (**b**) Calibration plot for Ag@Co-Al/PoPD nanohybrids. Pulse period: 0.1 s and amplitude: 0.025 V.

Differential pulse voltammetry: The differential pulse voltammograms of 4-NP at Ag@Co-Al/PoPD/GCE is displayed in Fig. 10. When increasing concentration of 4-NP, the 4-NP reduction peak current is also increased gradually and the linear concentration range is found to be 0.82 × 10^−9^ to 0.17 × 10^−5^ M (Ag@Co-Al LDH/PoPD). Figure 10b shows the calibration plot for 4-NP. The linear regression equation is found to be I_c_ (µA) = −7.9629 [4-NP] (µM) −1.7166 (R^2^ = 0.9990). From the linear regression equation it is found that Ag@Co-Al LDH/PoPD modified GCE shows better sensitivity and the details were given in Table 1. The DL was calculated from following equations with S/N ratio of 3.

$$DL=\frac{3s}{\sigma }$$

where σ is the slope of the calibration curve and S is the standard deviation. The estimated DL and quantification limit (QL) for the Ag@Co-Al LDH/PoPD are 63.7 nM and 0.2124 µM µA^−1^ respectively.

**Table 1:** Comparison of analytical performance of various electrochemical sensors for 4-NP, 2,4-DNP and UA,

Electrodes	Method	Analyte	Linear range (µM)	DL (µM)	Ref
HA-NP/GCE^a^	DPV	4-NP	1–300	0.60	^46^
Ag-Chitosan/GCE	SWV	4-NP	0.07–2.0	70 nM	^47^
Nano-Au/GCE	LSV	4-NP	10–100	8.0	^48^
Cu_2_O-sheets	DPV	4-NP	0.006–2.72	0.50	^49^
ZnO-Fe_2_O_3_ NP/Au/GCE	Amperometry	4-NP	1–10	0.83	^50^
MPS-poly(Vit-_B1_)^b^	CV	2,4-DNP	3–30	0.50	^51^
GO/MIPE^c^	DPV	2,4-DNP	1–150	—	^52^
HA film	calorimetry	2,4-DNP	2–600	0.70	^53^
MIPs/Ni fiber GCE	LC-MS	2,4-DNP	0.7–30	0.10	^54^
Pd@ Fe_2_O_3/_GCE	SWV	UA	0.96–107	0.41	^55^
SiO_2_@Au NPs@PANI^d^	DPV	UA	5–1100	2.0	^56^
GNPs^e^	SERS	UA	0–3.5*	0.11*	^57^
Poly(DPA) SiO_2_@Fe_3_O_4_/GCE^f^	DPV	UA	1.2–1.8	0.40	^58^
Ag@Co-Al/PoPD/GCE	DPV DPV DPV	4-NP 2,4-DNP UA	0.82 × 10^−9^ M–1.74 × 10^−6^ M 0.1 × 10^−6^ M–2.5 × 10^−6^ M 7.55 × 10^−7^ M –1.23 × 10^−5^ M	63.7 nM 50.2 nM 0.28 µM	This work

^a^HA-NP/GCE: Hydroxyapatite nanoparticle/glassy carbon electrode,

^b^MPS-Mesoporous silica,

^c^GO/MIPE-Grapheneoxide/ Molecularly Imprinted Polymer Electrode,

^d^PANI-Poly(aniline),

^e^GNPs-Gold nanoparticles,

^f^poly(DPA)-ploy(dipicolinic acid) and

*indicates millimolar/litre.

#### Electrochemical sensing of 2,4-dinitrophenol

**Figure 11:**
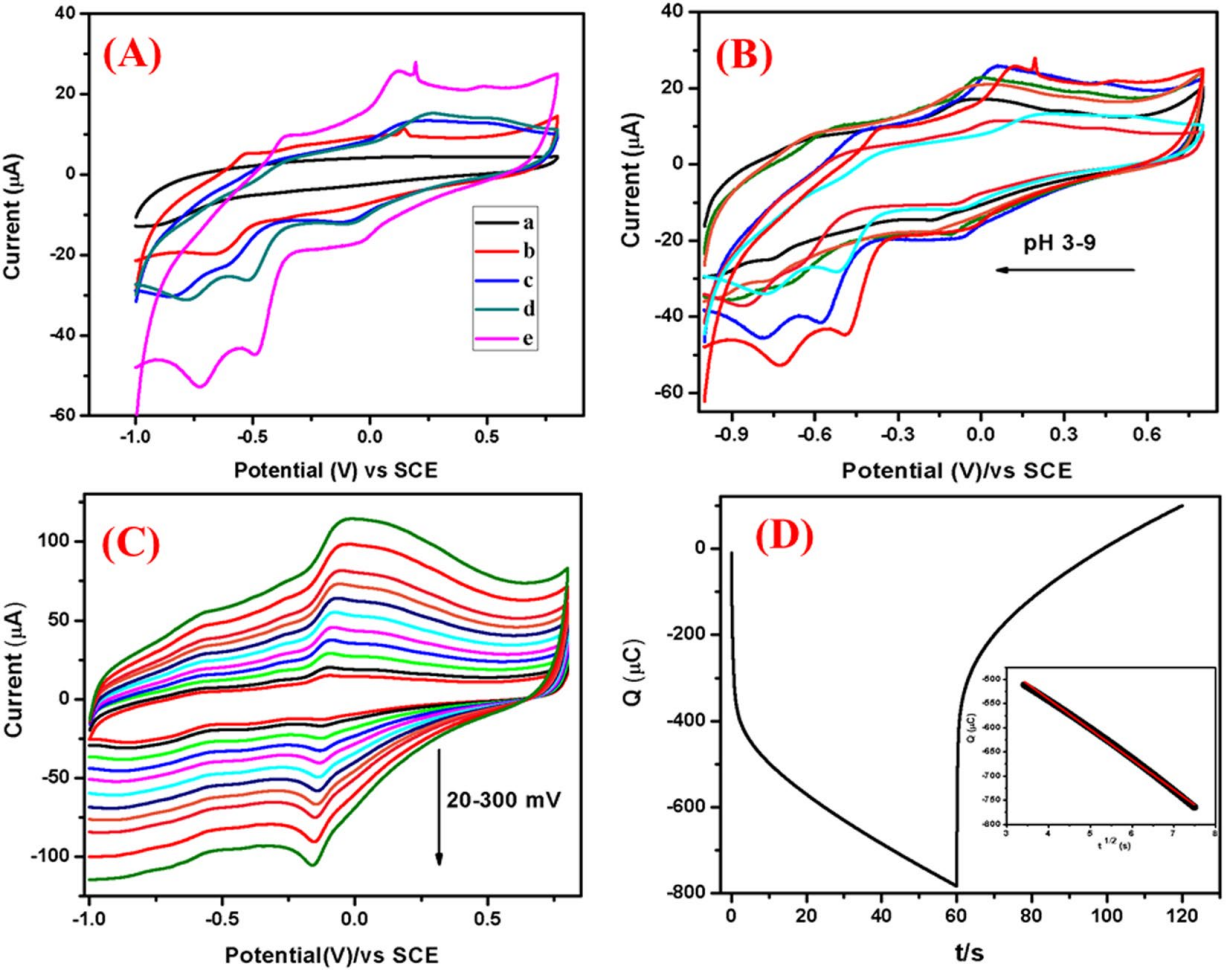
(**A**) Cyclic voltammograms of (a) bare GCE (Black), Ag@Co-Al/PoPD/GCE (Red) in absence of 2,4-DNP, Co-Al/GCE (Blue), Co-Al/PoPD/GCE (Green) and Ag@Co-Al/PoPD/GCE (Pink) in the presence of 1 × 10^−3^ M 2,4-DNP (pH 5) at the scan rate of 50 mVs^−1^. (**B**) Effect of pH (pH 3–9) (**C**) effect of scan rates (20–300 mVs^−1^) of Ag@Co-Al/PoPD/GCE, (**D**) Chronocoulometric curve of Ag@Co-Al/PoPD/GCE (inset fig: t^1/2^ vs Q).

Electrochemical sensing of DNP: The CVs of 2,4-DNP at bare GCE, Co-Al LDH/GCE, Co-Al LDH/PoPD/GCE and Ag@Co-Al LDH/PoPD/GCE in absence and presence of 1 × 10^−3^ M 2,4-DNP in (pH = 5) at the scan rate of 50 mVs^−1^ is shown in Fig. 11A. The voltammograms in the absence of 2,4-DNP shows one cathodic peak at 0.14 V for Ag@Co-Al LDH/PoPD/GCE. The presence of 2,4-DNP gives two anodic and three cathodic peaks for Co-Al LDH/GCE, Co-Al/PoPD/GCE and Ag@Co-Al LDH/PoPD. Bare GCE shows these peaks at higher potentials with low current when compared to that of the modified electrodes. This clearly demonstrates the strong electrocatalytic effect of the modified electrodes. The order of reduction peak potential and current of 2,4-DNP can be given as, pure Co-Al LDH [−0.85 V, −30.85 µA] < Co-Al/PoPD [−0.78 V, −31.37 µA] < Ag@Co-Al/PoPD [−0.72 V, −53.11 µA]. From this, it is clear that the Ag@Co-Al LDH/PoPD/GCE exhibits greater electrochemical sensing behaviour towards the redox reaction of 2,4-DNP when compared to bare GCE. The electrochemical sensing behaviour of Ag@Co-Al LDH/PoPD/GCE may attribute to the modifying layer that have higher surface active owing to the presence of nanoparticles as evident from HR-TEM images and the presence of –OH group that favours the formation of hydrogen bonding between 2,4-DNP and the modifying layer.

The redox pathway of 2,4-DNP at Ag@Co-Al LDH/PoPD/GCE occurs via simultaneous substitution of the hydroxyl radical by replacing eliminating two nitro groups which gives (+0.11 and +0.47 V) the product (I)^43^. The quinone further reduced as product (II) (−0.06, −0.49 and −0.72 V) and all the nitro groups are reduced to amine groups^44^.

**Figure 12:**
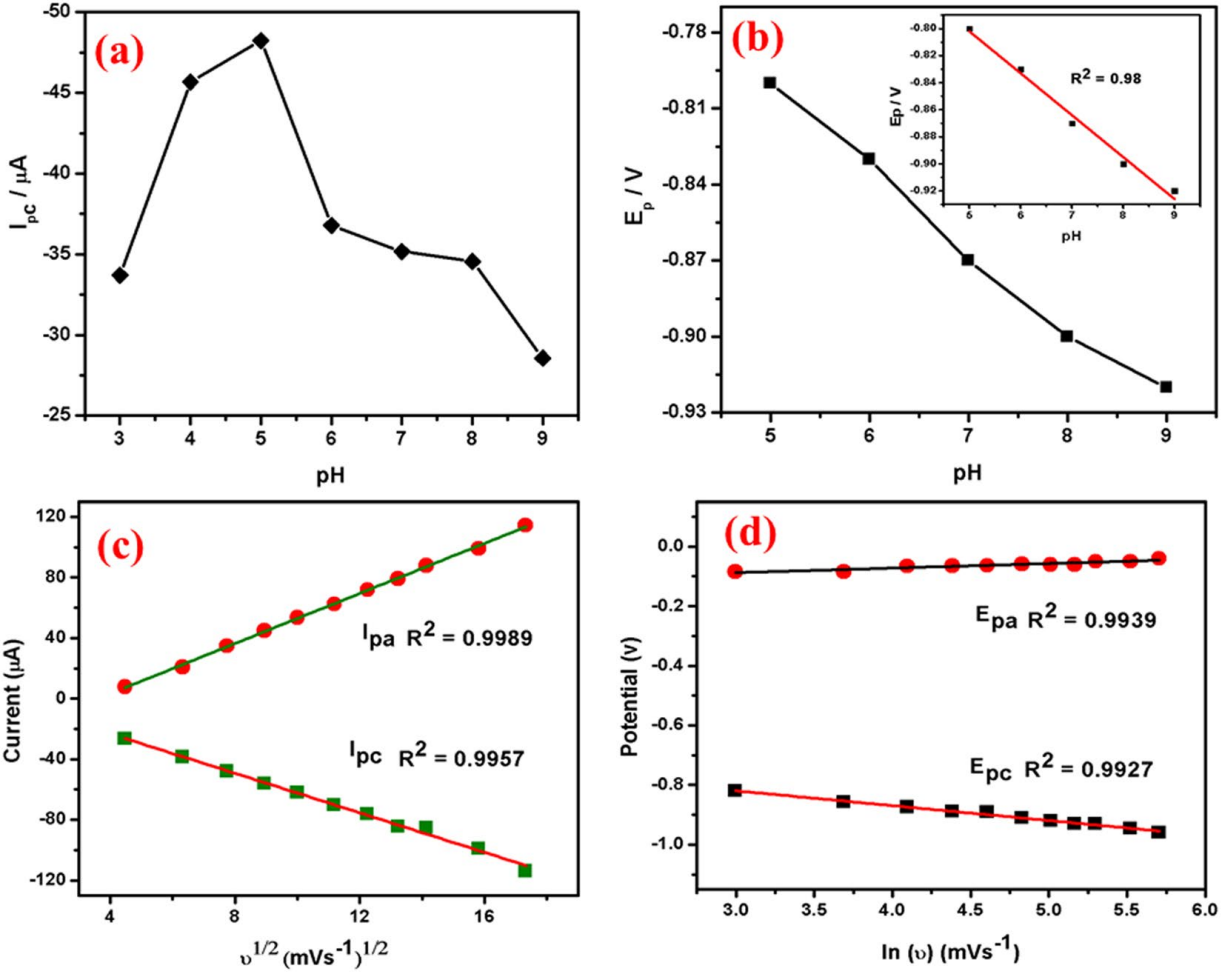
(**a**) pH vs Ip and (**b**) pH vs Ep, (c) The derivative plot of ʋ^1/2^ vs Ip and (**d**) ln(ʋ) vs Ep for Ag@ Co-Al/PoPD in 1 mM 2,4-DNP (pH = 5).

Effect of pH: Figure 11B shows effect of pH (pH 3–9) on the electrochemical response at 1 × 10^−3^ M 2,4-DNP at Ag@Co-Al LDH/PoPD modified GCE. The reduction peak current of 2,4-DNP increased when increasing pH from 3 to 9. The peak current is high at pH 5 and thus it was chosen for further electrochemical studies. In case of higher pH, the current response was less. The reason for the higher reduction current of 2,4-DNP at pH 5 may be attributes to the H-bonding interaction between nitro groups of 2,4-DNP with the modifying layer. Figure 12(a,b) shows the plot of pH vs I_p_ and pH vs E_p_ plots for Ag@Co-Al LDH/PoPD/GCE in 1 × 10^−3^ M 2,4-DNP.

Effect of Scan rate: To study the kinetic behaviour of 2,4-DNP at Ag@Co-Al LDH/PoPD/GCE, effect of scan rates were investigated in the range of 30–300 mVs^−1^ (Fig. 11C). Figure 12c shows the plot of square root of scan rate versus I_p_, for Ag@Co-Al/PoPD/GCE. The linear regression equation of the plot is given as,

$${{\rm{I}}}_{{\rm{pc}}}=0.3185{({\rm{\upsilon }})}^{1/2}-0.6427\,({{\rm{R}}}^{2}=0.9957)$$

From the slope value (> 0.5) the electrode surface is adsorption controlled process. The adsorption electrode process behaviour was may be attributed to the presence of two nitro groups present in 2,4-DNP, which binds effectively on the surface through H-bonding. From the relationship between ln(υ) vs E_p_ (Fig. 12d) the linear regression equations is obtained as,

$${{\rm{E}}}_{{\rm{pc}}}=-\,0.6805\,\mathrm{ln}({\rm{\upsilon }})-0.1904\,({{\rm{R}}}^{2}=0.9927)$$

Chronocoulometry studies: Diffusion coefficient (D) value was calculated by using chronocoulometry technique for the modified nanohybrids electrodes (Fig. 11D). The plot of the square root of time (t^1/2^) against charge (Q) showed a linear relationship (inset in Fig. 11D). By considering geometric area (A) = 0.07 cm^2^, number of electrons (n) = 8, and Concentration (c) = 0.001 M. The calculated diffusion coefficient value is 4.07 × 10^−12^ cm^2^ s^−1^ for Ag@Co-Al LDH/PoPD/GCE.

**Figure 13:**
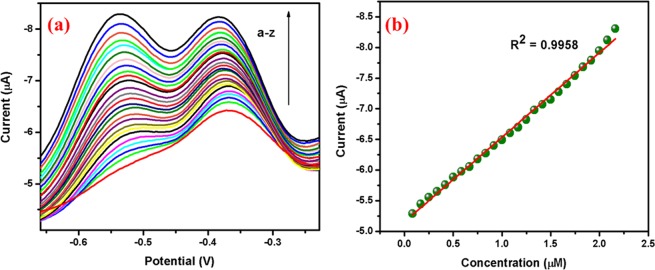
(**a**) Differential pulse voltammograms of different concentrations (from a to z: 5 µM–130 µM) of 2,4-DNP at Ag@Co-Al/PoPD/GCE and (**b**) Calibration plot of 2,4-DNP at Ag@Co-Al/PoPD/GCE. Pulse period: 0.1 s and amplitude: 0.025 V.

Differential pulse voltammetry: The DPV response of 2,4-DNP using Ag@Co-Al LDH/PoPD/GCE is displayed in Fig. 13a. The increase of 2,4-DNP concentration, significaly increases reduction peak current of 2,4-DNP. The linear range and sensitivity was found to be 0.1 × 10^−6^ to 2.5 × 10^−6^ M and 1.73 µM µA^−1^ respectively. The linear regression equation is found as I_c_ (µA) = −5.3229 [DNP] (µM)−1.0301 (R^2^ = 9958). The obtained sensitivity value implies that the Ag@Co-Al/PoPD LDH/GCE is highly sensitive towards DNP. The estimated DL and quantification limit for the Ag@Co-Al LDH/PoPD/GCE is 50.2 nM and 0.1676 µM µA^−1^ respectively.

#### Electrochemical sensing of uric acid

**Figure 14:**
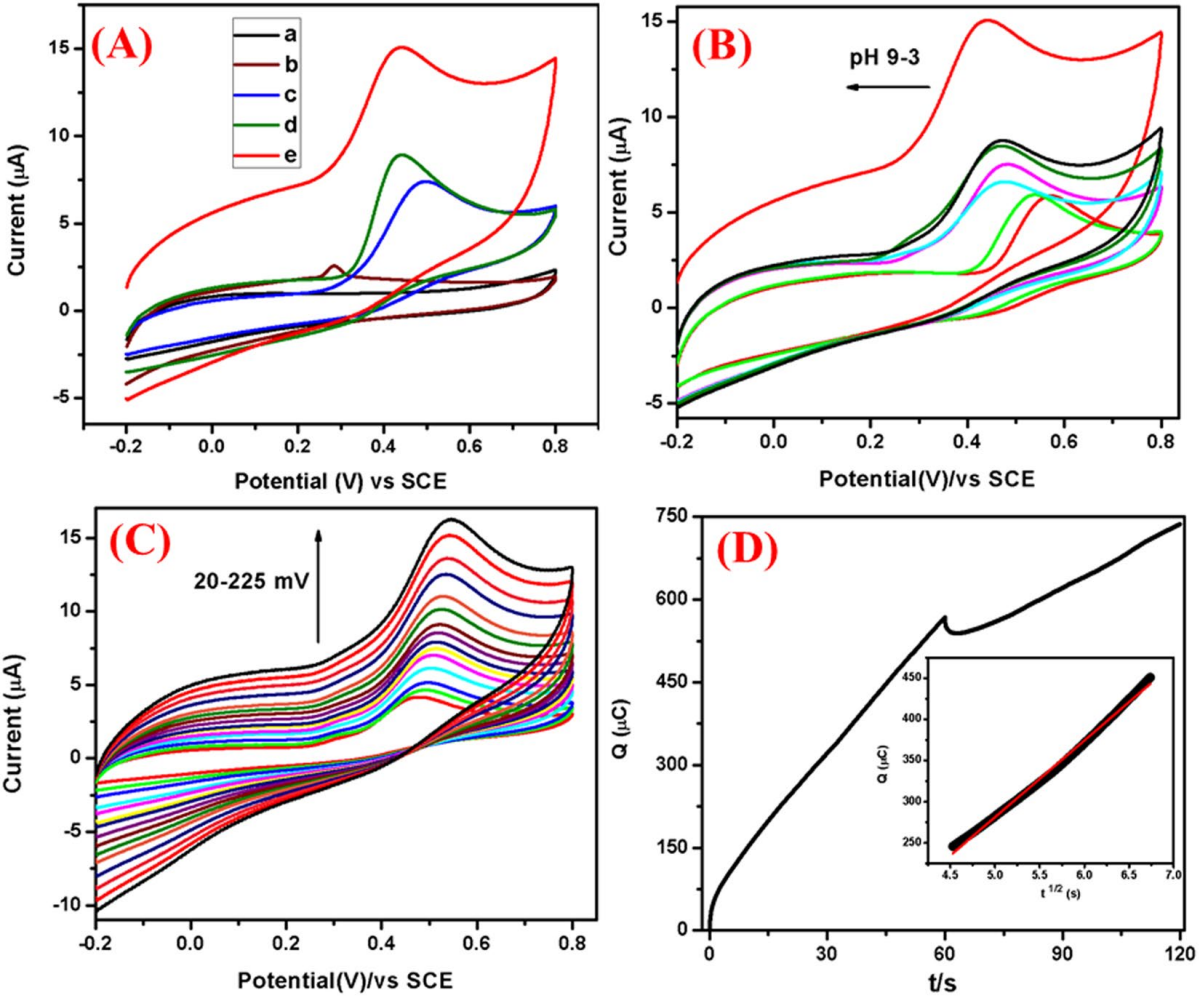
(**A**) Cyclic voltammetry of (a) bare GCE (Black), Ag@Co-Al/PoPD/GCE (brown) in absence of 1 mM UA, Co-Al/GCE (Blue), Co-Al/PoPD/GCE (Green) and Ag@Co-Al/PoPD/GCE (Red) in the presence of 1 × 10^−3^ M UA (pH 7) at the scan rate of 50 mVs^−1^. (**B**) pH effect of Ag@Co-Al/PoPD/GCE (pH 3–9), (**C**) Scan rate at 20–225 mV, (**D**) Chronocoulometry (inset fig: t^1/2^ vs Q).

Cyclic voltammetry of UA: Fig. 14A displays the cyclic voltammograms of bare GCE, pure Co-Al LDH/GCE, Co-Al/PoPD/GCE and Ag@Co-Al/PoPD/GCE in blank PBS (0.1 M PBS, pH = 7.0) at the scan rate of 50 mVs^−1^. No reduction peaks observed for bare GCE and pure Co-Al LDH/GCE, certainly in presence of Ag@Co-Al LDH/PoPD modified GCE gives the significant peaks in the potential range of −0.2 to +0.8 V. Further dynamic property of Ag@Co-Al LDH/PoPD/GCE examined by variation of scanning rates. The scan rate is directly proportional to the anodic current it suggesting charge-transfer controlled process. Figure 14(A–E) depicts the scan rate of Ag@Co-Al/PoPD/GCE in the absence and presence of 1 × 10^−3^ M UA at of 50 mVs^−1^. All the peaks value were tabulated in SI Table 1. The modified electrodes showed enhanced anodic peak current than the bare GCE with shifted peak potential indicates the strong electrochemical sensing ability of the modified electrodes. These observed electrochemical sensing behaviours ascribed to (i) greater active surface area of modifying layer (ii) The free intercalated and surface –OH group as strong evident by FTIR studies. Moreover, LDHs have been the hydrogen bond acceptor strength with the polymer group. Aforementioned reasons may be making easier to oxidize the UA at GCE modified electrode surface. Apart from, free surface –OH group interact with the carbonyl group of UA forms hydrogen bonding. Thus weakening of hydroxyl bond to facilitate the electron transfer through (hydroxyl group of UA) O-H----O (surface hydroxyl group of Ag@Co-Al LDH/PoPD). The mechanism of UA based on the above discussion and the following steps can derive the previous reports^﻿﻿45﻿﻿^: (a) In the first step, modified electrodes interact with UA via mass transport by diffusion. (b) in second step, the UA adsorbed on Ag@Co-Al LDH/PoPD/GCE. (c) Finally, adsorbed UA undergoes internal electron transfer with the formation of oxidized product of UA.

Effect of pH: The pH effect on electrochemical response of 1 × 10^−3^ M UA in the pH range of 3–9 is shown in Fig. 14B. The UA oxidation peak current increases gradually with increasing pH from 3 to 9, the pH 7 was chosen as the optimised pH. In case of higher pH, the current response decreased so pH 7 chosen for further analysis. However, the reason for the higher oxidation current at pH 7 attributes to the interaction of nitrogen and carbonyl groups of UA with the modified electrode surface. Figure 14(A,B) shows the plot of pH vs Ip and pH vs Ep plots for Ag@Co-Al LDH/PoPD/GCE in 1 × 10^−3^ M UA.

**Figure 15:**
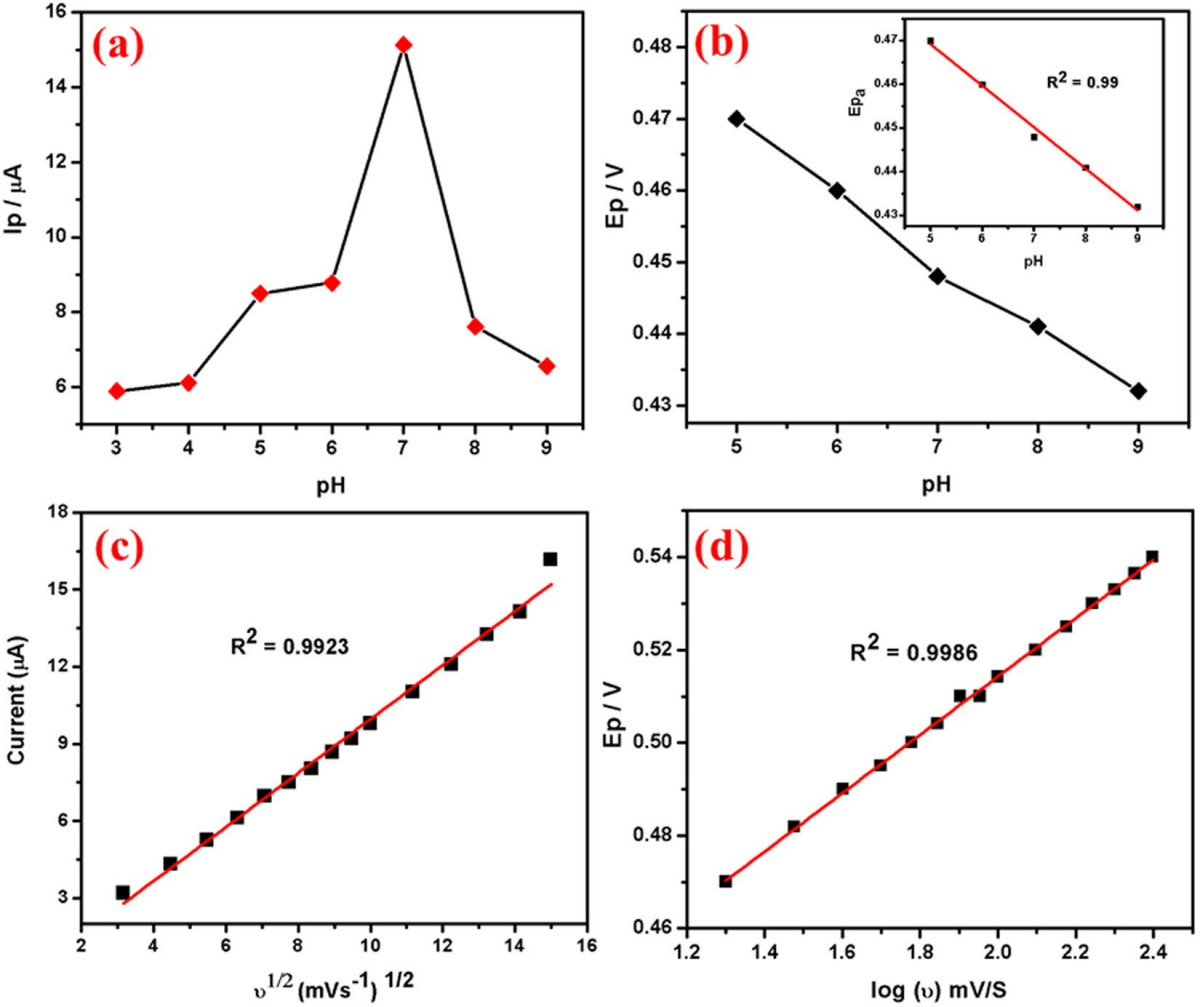
The plot of (**a**) pH vs Ip and (**b**) pH vs Ep, (**c**) The derivative plot of ʋ^1/2^ vs Ip and (**d**) ln(ʋ) vs Ep for Ag@ Co-Al/PoPD in 1 mM UA (pH 7).

Effect of scan rate: Figure 14C reveals that the oxidation peak current moved positively with increasing scan rate from 20–225 mVs^−1^ (pH 7). It suggests that the modified electrode has well electrochemical property and fast electron transfer ability. Further, the obtained scan rate slope is less than 0.5, so the electrode process is diffusion controlled and the number of electrons involved in UA oxidation (Eq. 4.3) is calculated as 1.91 (~2). The plots of (υ)^1/2^ vs Ipa and log(υ) versus log(Epa) are shown in Fig. 15(c,d) and the obtained linear regression equations were given as,

$${\rm{Ipa}}=-\,0.1254\,{({\rm{\upsilon }})}^{1/2}+0.5464\,({{\rm{R}}}^{2}=0.9923),$$

and

$${\rm{Epa}}=-\,0.3884\,\log ({\rm{\upsilon }})+0.0629\,({{\rm{R}}}^{2}=0.9986)$$

Chronocoulometry: Diffusion coefficient (D) was investigated using Chronocoulometry for Ag@Co-Al/PoPD/GCE nanohybrids and shown in Fig. 14D. (inset: Fig. 14D the background subtraction, Q vs t^1/2^ showed a linear relationship). By substituting A = 0.07 cm^2^, n = 2, and c = 0.001 M. The calculated diffusion coefficient for Ag@Co-Al/PoPD/GCE nanohybrids is 4.13 × 10^−12^ cm^2^ s^−1^.

**Figure 16:**
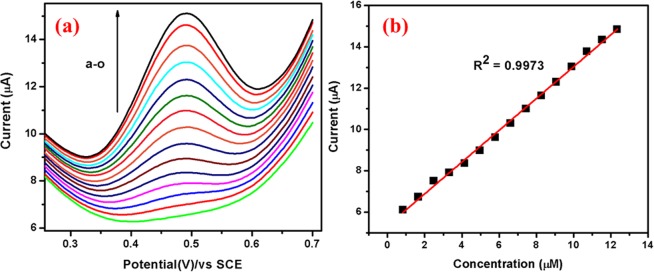
(**a**) Differential pulse voltammograms of different concentrations (from a to o: 50 µM–750 µM) of UA at Ag@Co-Al/PoPD/GCE and (**b**) Calibration plot for Ag@Co-Al/PoPD nanohybrids. Pulse period: 0.1 s and amplitude: 0.025 V.

Differential pulse voltammogram of UA: The sensitivity and DL of Ag@Co-Al LDH/PoPD/GCE towards UA was further determined by DPV. Figure 16a displays the DPV’s of UA oxidation at Ag@Co-Al LDH/PoPD/GCE in 0.1 M PBS. The oxidation peak current increases gradually with increasing the concentration of UA. The calibration plot for UA is shown in Fig. 16b. The linear range is found between 7.5 × 10^–7^–1.2 × 10^−5^ M). The calculated DL and QL of Ag@Co-Al LDH/PoPD/GCE are 0.289 µM, and 0.9717 µM µA^−1^ respectively. From the experiment, it is clear that the Ag@Co-Al LDH/PoPD/GCE has relatively high sensitivity towards oxidation of UA.

#### Reproduceablity, stability and inference study

In order to investigate reproducibility of Ag@Co-Al/PoPD/GCE, five modified electrodes were fabricated. The response of 1 × 10^−3^ M 4-NP and 2,4-DNP was measured for five modified electrodes and the relative standard deviation (RSD) was found to be 2.85%, (4-NP) and 2.80% (2,4 DNP). These RSD valuse was clearly indicates the prepared modified electrode has good reproducibility.

To find stability, Ag@Co-Al/PoPD/GCE was repeatedly used to measure 1 × 10^−3^ M 4-NP, 2, 4-DNP for 20 days. The modified electrode was kept in refrigerator at 5 °C when not in use. The cathodic peak current of 4-NP and 2,4-DNP deduced to 97% and 96% respectively after seven days, further, to 89% and 86% after 20 days. The modified electrode was further studied in presence of higher concentrations of interfering species (100 fold excess of 4-NP). A 50 fold excess of K^+^, Na^+^, Mg^2+^, Ca^2+^, Cl^−^, SO_4_^2−^, and NO_3_^−^ was not interfere in the determination of 4-NP and 2,4-DNP. However, the 25 fold excess of Zn^2+^, Cu^2+^ and Fe^2+^ ions interfered in the determination of 4-NP and 2,4-DNP (i.e., the reduction peak current decreases by ~5% and there is no change in the peak potential). Also, 10 fold excessive concentration of 2-nitrophenol, 3-nitrophenol, 4-chlorophenol, 2-chlorophenol interfere in the determination of 4-NP and 2,4-DNP. These results suggest that the Ag@Co-Al LDH/PoPD/GCE can be applied for real sample analysis.

In addition, effect of interfering species (dopamine) on UA determination was also studied by using Ag@Co-Al LDH/PoPD/GCE. It was found the equimolar concentration of dopamine do not interfere with UA determination.

#### Analytical applications

To evaluate the proposed Ag@Co-Al LDH/PoPD/GCE for the analytical applications, DPV techniuqes is used to determine the concentrations of 4-NP, 2,4-DNP and UA in real sample solutions. The standard addition method (Tables 2 and 3) was used to determine concentration of these analytes. The real water samples were collected from different places (Chennai, Trichy, Madurai and Tirunelveli) in Tamilnadu. The table values conclude the overall results observed in the determination of 4-NP, 2,4-DNP and UA in the four independent solutions. The recoveries of 4-NP, 2,4-DNP and UA determination by Ag@Co-Al LDH/PoPD/GCE is 96.0–101.2%, 97.0–100.2% and 98.5–101.5% respectively. These results suggest that Ag@Co-Al LDH/PoPD/GCE could be use for the determination of nitroaromatics compounds and biomolecules in real samples.

**Table 2:** Determination of 4-NP and DNP in water.

Samples	Added (µM)		By This Method (µM)		RSD (%)		Recovery (%)	
	4-NP	DNP	4-NP	DNP	4-NP	DNP	4-NP	DNP
I	5.0	5.0	4.92	4.85	2.85	2.80	98.4	97.0
II	5.0	5.0	5.06	4.90	3.35	2.95	101.2	98.0
III	5.0	5.0	4.80	4.95	4.05	3.10	96.0	99.0
IV	5.0	5.0	4.88	5.01	3.70	3.72	97.6	100.2

**Table 3:** Determination of Uric Acid.

Samples	Detected (µM)	Added (µM)	RSD (%)	Recovery (%)
I	17.9	25.0	2.3	100.6
II	20.4	25.0	2.4	101.5
III	12.0	25.0	3.4	98.5
IV	11.7	25.0	3.1	99.4

## Conclusion

In conclusion, we have developed a simple method to synthesise Ag decorated PoPD reinforced Co-Al LDH. The resulting materials were investigated using several characterizations techniques such as XRD, Raman, FT-IR, DRS-UV vis, PL, TGA, FESEM and HR-TEM analysis. The rapid modification process, greater sensitivity and lower detection limits are the key attractive features of Ag@Co-Al LDH/PoPD/GCE. The electrochemical sensor delivered good recovery of 4-NP, 2,4-DNP and UA in different real samples. Hence, Ag@Co-Al LDH/PoPD/GCE will become great potential in the field of electrochemical sensor.

## Supplementary information

Fabrication of Ag@Co-Al Layered Double Hydroxides Reinforced poly(o-phenylenediamine) Nanohybrid for Efficient Electrochemical Detection of 4-Nitrophenol, 2,4-Dinitrophenol and Uric acid at Nano Molar
