# Supplementary material for: Fabrication of Ag@Co-Al Layered Double Hydroxides Reinforced poly(o-phenylenediamine) Nanohybrid for Efficient Electrochemical Detection of 4-Nitrophenol, 2,4-Dinitrophenol and Uric acid at Nano Molar Level
Source: Sci Rep. 2019 Sep 13;9:13250. doi: 10.1038/s41598-019-49595-y (PMC6744444; doi:10.1038/s41598-019-49595-y)
Supplement: Supplementary file 1 — Fabrication of Ag@Co-Al Layered Double Hydroxides Reinforced poly(o-phenylenediamine) Nanohybrid for Efficient Electrochemical Detection of 4-Nitrophenol, 2,4-Dinitrophenol and Uric acid at Nano Molar [file 41598_2019_49595_MOESM1_ESM.docx]

**Supporting Information**

**Fabrication of Ag@Co-Al Layered Double Hydroxides Reinforced poly(o-phenylenediamine) Nanohybrid for Efficient Electrochemical Detection of**

**4-Nitrophenol, 2,4-Dinitrophenol and Uric acid at Nano Molar Level**

**T. Dhanasekaran^1,2^, R. Manigandan^3^, A. Padmanaban^1^, R. Suresh^4^, K. Giribabu^5^ and
 V. Narayanan^1 *^**

*^1^Department of Inorganic Chemistry, University of Madras, Chennai, India*

*^2^National Centre for Sustainable Coastal Management, Anna University Campus,*

*Chennai, India*

*^3^School of Electronic Science and engineering, University of electronic Science and Technology of China, Chengdu, China*

*^4^Department of Analytical and Inorganic Chemistry, University of Concepcion, Chile*

*^5^Electrodics and Electrocatalysis Division, CSIR-CECRI, Karaikudi, India*

*Email: vnnara@yahoo.co.in*

**Instrumentation**

The crystal structure, and crystalline size of synthesized samples were characterized by Seifert X-ray powder diffractometer using Cu k_α1_ radiation (λ= 1.5406 Å). The Raman spectra of samples were recorded by using BRUKER RFS 27: Stand-alone FT-Raman spectrometer equipped with Nd: YAG 1064 nm. XPS analysis was performed using Thermo Scientific K-alpha (180-200 W). The functional groups were analyzed from FTIR spectroscopy by Schimadzu FT-IR 8300, the wave number range is 400 to 4000 cm^-1^. Pellets of samples from milligram of each sample were diluted with 100 mg of vacuum-dried IR-grade KBr and pressure of 10 tons. Thermogarvimetric Analysis was carried out using thermal analysis instrument (SDT Q600), under flow of UHP nitrogen or argon gas (flow rate = 100 cm^3^ min^-1^) as protective gas using an alumina crucible. The heating rate of 10 °C min^-1^ in nitrogen atmosphere (~2 mg) analyzed at room temperature. The optical property was measured by using Perkin Elmer lambda 650 spectrophotometer. The emission of photoluminescence (PL) spectra were recorded using a Perkin-Elmer LS5B luminescence spectrophotometer. The surface morphological features were examined using FESEM and HR-TEM. Zeiss field emission scanning electron microscope operating at 3 kV and HR-TEM used as FEI TECNAI T20 G_2_ instrument for analyzed low and high magnification images operating at an accelerating voltage of 200 kV. Carbon coated TEM grids (200 Mesh Type-B) were purchased from Ted Pella Inc. USA. The electrochemical experiments were carried out by using CHI 1103A electrochemical workstation with three-electrode system. The platinum wire was a counter electrode, while bare and modified GCE as a working electrode and saturated calomel was used as a reference electrode respectively.

**Experimental**

**Synthesis of Co-Al Layered Double Hydroxides**

To the aqueous solution of 0.06 M cobalt nitrate and 0.02 M aluminium nitrate (mole ration 1:3), a freshly prepared urea (0.5 M) solution was added slowly. Then 0.6 M NaOH aqueous solution was added drop wise into the above solution. The resulting suspension was transferred into Teflon lined auto-clave and kept in a muffle furnace at 160 °C for 14 h. After that, the suspension was cooled to room temperature. Finally, the obtained product was centrifuged at 6000 rpm and washed many times using double distilled (DD) water-ethanol (50:50) mixture and dried at 60 °C overnight in hot air oven.

**Synthesis of Poly(o-phenylenediamine)**

The monomer, o-PD (1.0 g, 9.25 mmol) was dissolved in 25 ml of hydrochloric acid (1 M HCl) solution. A 10 ml of freshly prepared ammonium persulfate (1.5 g, 6.57 mmol) aqueous solution was added drop by drop to the above solution. The colour of reaction medium changes from pink to brown. The reaction is performed under stirring condition for 12 h at 30 ^o^C. An orange-red precipitate washed with water many times. At last PoPD powder was dried in a vacuum oven at 60 ^o^C for 6 h.

**Synthesis of Poly(o-phenylenediamine) supported Co-Al Layered Double Hydroxides**

To synthesize PoPD/Co-Al LDH, 250 mg of LDHs was suspended in 100 ml water. To this solution, 50 mg of freshly PoPD dispersion (10 ml) in methanol was added drop by drop into the solution. The solution was transferred into Teflon-walled steel autoclave vessel and was aged at 120 ^o^C for 8 h. The obtained product was washed with DD water and dried at 60 ^o^C in a hot air oven overnight.

**Synthesis of Ag@PoPD reinforced Co-Al Layered Double Hydroxides**

To synthesize Ag@PoPD/Co-Al LDH, 100 mg of PoPD/LDHs was suspended in 250 ml double necked round bottom flask using Milli-Q water with magnetic stirring. The freshly prepared 169 mg of silver nitrate (1 mM) solution (30 ml) was added drop by drop. The reaction mixture was constantly stirred for 12 h under N_2_ atm at room temperature. The suspension was centrifuged at 6000 rpm and washed several times using DD water. Finally the product is dried in a vacuum oven at 40 ^o^C for 12 h.





SI.1 Fig. Raman spectra of synthesis of Co-Al LDH (black), Co-Al/PoPD (red),
Ag@Co-Al LDH/PoPD (blue)


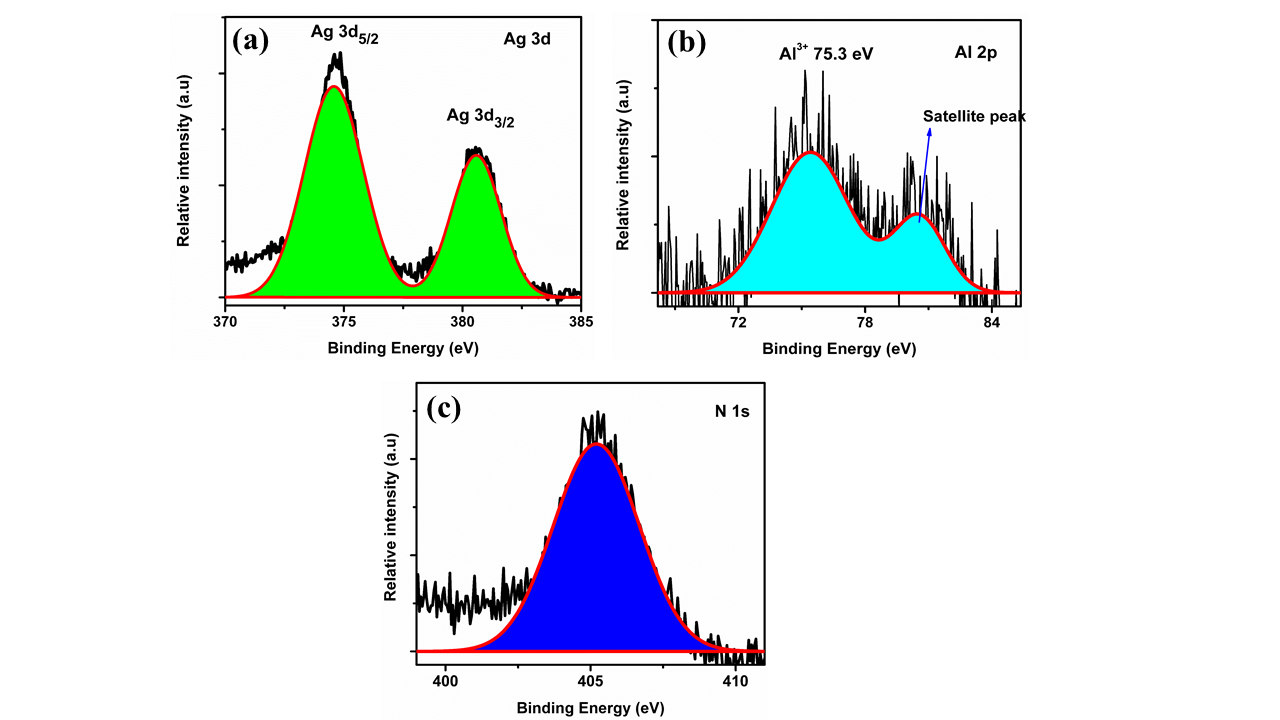


SI. 2 (a-c) XPS core level spectrum of Ag 3d, Al 2p and N 1s

**Photoluminescence and Thermogravimetric analysis**

The photoluminescence (PL) spectra of Co-Al LDH, Co-Al LDH/PoPD and Ag@PoPD/Co-Al LDH materials were given in SI Fig. 3. The PL spectra of Co-Al LDH show the emission bands in the range of 350-600 nm at λ_exc_ = 350-480 nm [1]. The Co-Al/PoPD gives two emission bands at 415 and 439 nm which correspond to PoPD and Co-Al LDH respectively. Furthermore, the PL spectrum of Ag@Co-Al LDH/PoPD displays bands at 413 nm and 435 nm with greater luminescence intensity compared to that of Co-Al LDH. This might be due to existence of Ag nanoparticles in Ag@Co-Al LDH/PoPD sample.

The thermo gravimetric curves of Co-Al LDH, Co-Al LDH/PoPD and Ag@PoPD/Co-Al LDH were obtained by using TGA. The thermogram of pure Co-Al LDH (SI Fig. 3b) shows three-step weight loss. The ﬁrst weight loss (50–110 ºC) step is due to loss of intercalated of water molecules present in Co-Al LDH and the weight loss at around 150-200 ºC is attributed to removal of hydroxyl group from the surface of Co-Al LDH. The third weight loss occurs at 285-380 ºC which is due to conversion of metal hydroxides into their oxides [2]. The thermogram of Co-Al LDH/PoPD (SI Fig.3b) nanohybrid showed decomposition with multi-step weight losses. The ﬁrst step (50–120 ºC) indicates the loss of water from Co-Al LDH/PoPD sample. The weight loss steps occur between 150-195 and 290-330 ºC which might be attributed to loss of PoPD matrix and conversion of metal hydroxides into their oxides. These multi-decomposition steps indicated that there is probably some interaction exist between metal hydroxide particles and PoPD backbone [3]. The thermogram of Ag@Co-Al LDH/PoPD (SI Fig.3b) displays three steps weight losses at 50-120, 165-200 and 270-335 ºC. The first two steps correspond to the removal of water molecules and the third one corresponds to decomposition of PoPD respectively. Eventhough the TGA was performed under nitrogen atmosphere, the residual mass increase in Co-Al LDH/PoPD was observed that might be due to formation of metal oxide particles in the PoPD matrix.


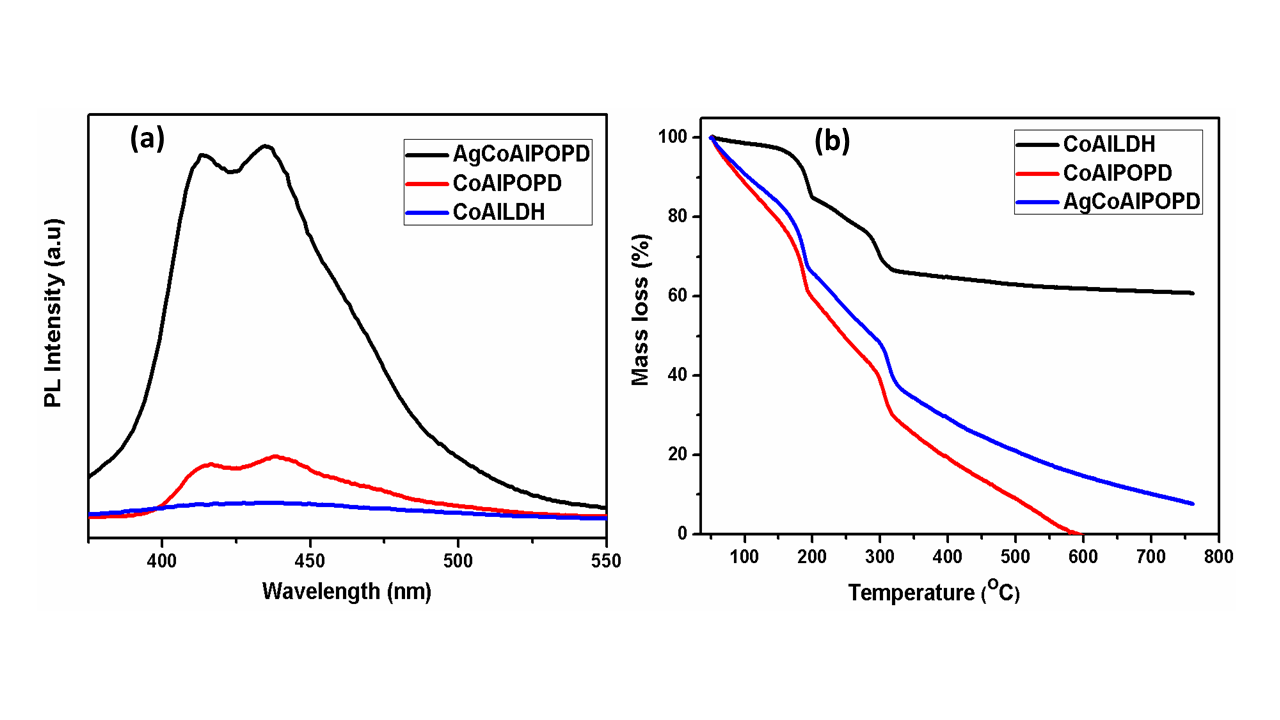


SI. Fig. 3 (a) Photoluminescence spectra of Co-Al LDH (blue), Co-Al LDH/PoPD (red),
Ag@Co-Al LDH/PoPD (black), (b)Thermogram of Co-Al LDH (black), Co-Al LDH/PoPD (red), Ag@Co-Al LDH/PoPD (blue).


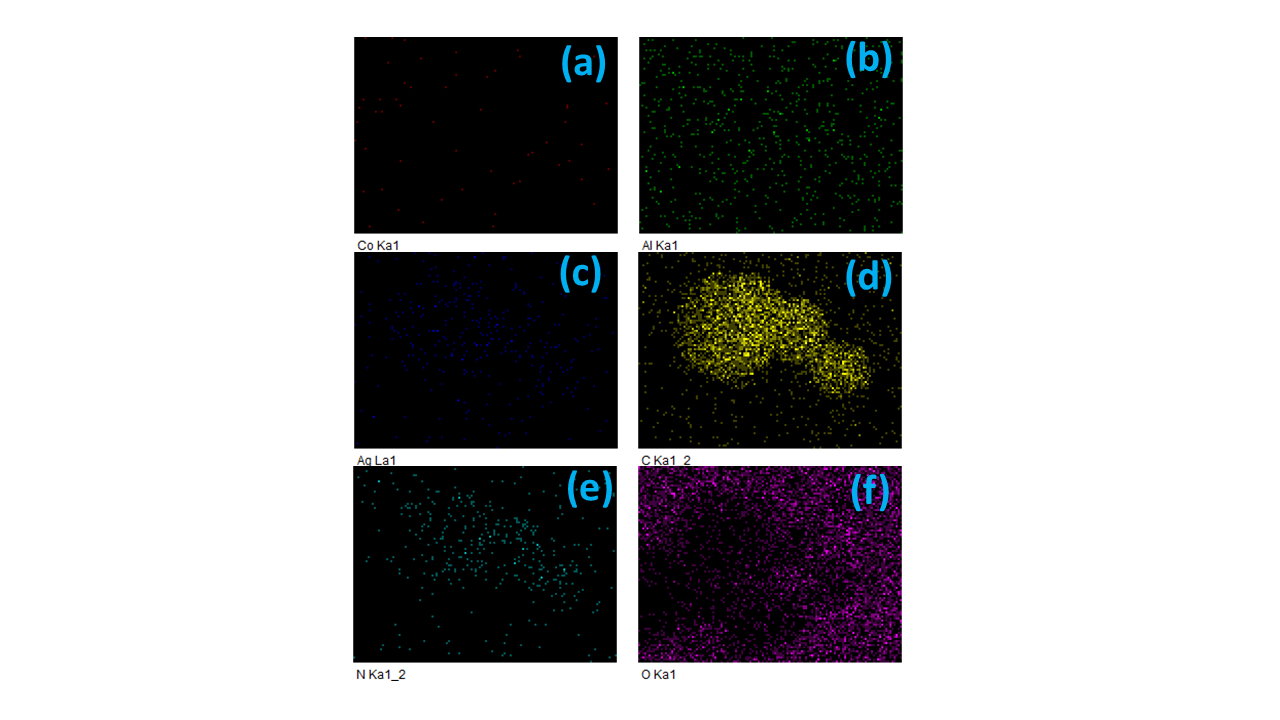


SI Fig. 4 (a-f) shows the elemental mapping (a) Co, (b) Al, (c) Ag, (d) C, (e) N, (f) O for Ag@Co-Al LDH/PoPD nanohybrids.


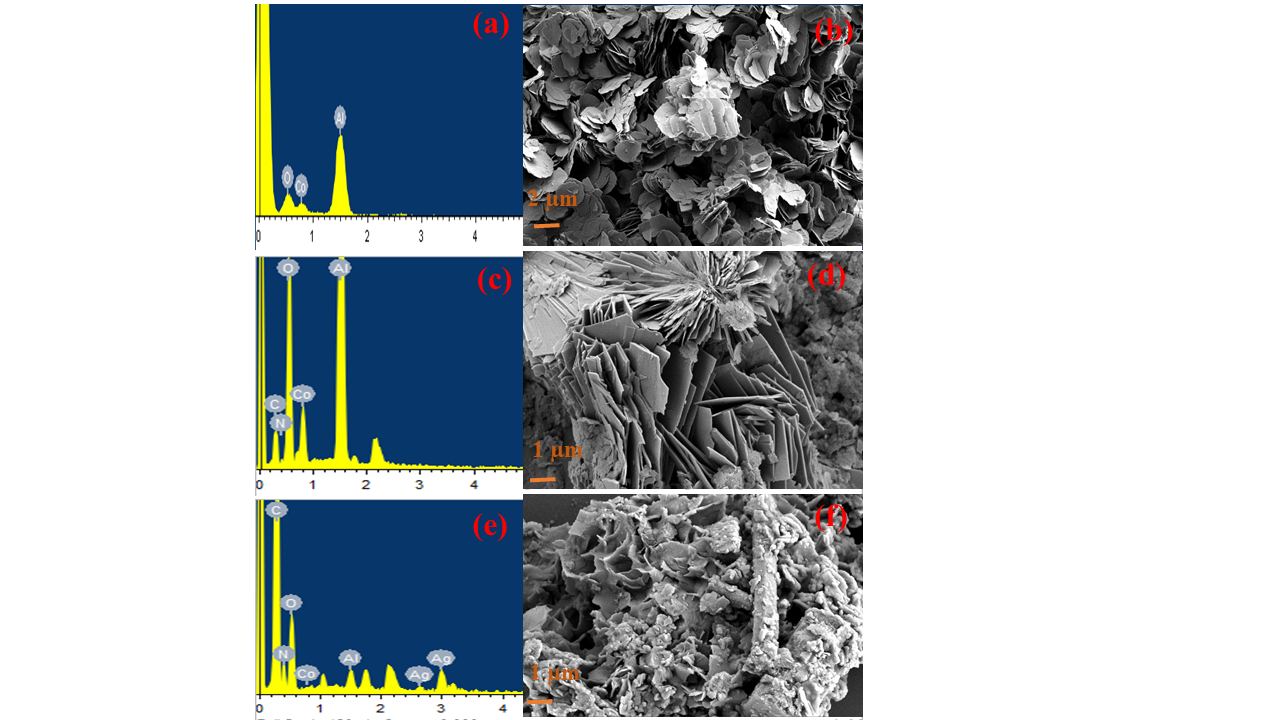


SI. Fig. 5 (a,c,e) EDAX spectrum of pure Co-Al LDH, Co-Al LDH/PoPD and Ag@Co-Al LDH/PoPD, (b.d.f) FESEM images of Co-Al LDH, Co-Al LDH/PoPD and Ag@Co-Al LDH/PoPD.


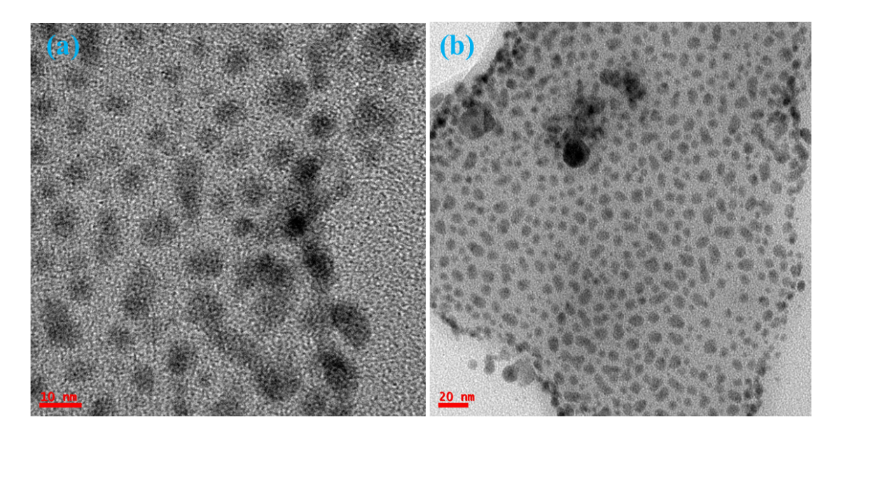


SI. Fig. 6 (a&b) HRTEM magnified images of the Ag@Co-Al LDH/PoPD nanohybrids.


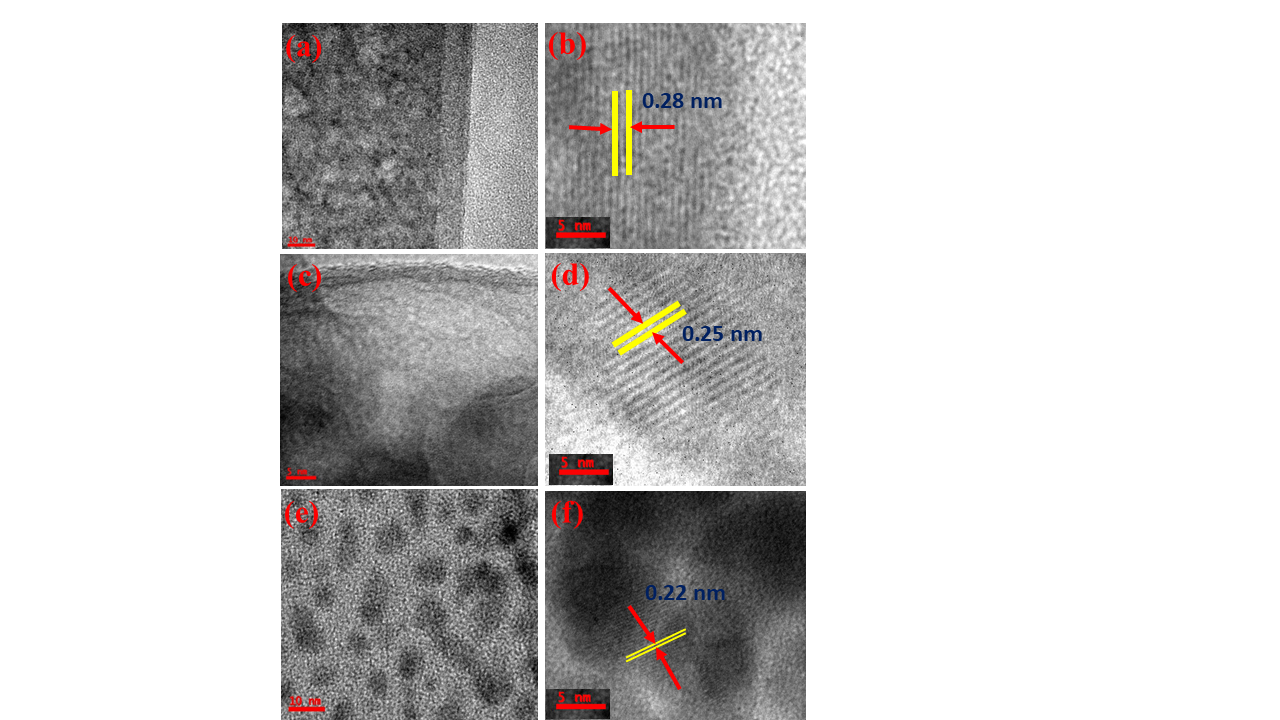


SI. Fig.7 (a,c,e) high magnification HRTEM images of pure Co-Al LDH, Co-Al LDH/PoPD and Ag@Co-Al LDH/PoPD, (b.d.f) Fringes pattern of Co-Al LDH, Co-Al LDH/PoPD and Ag@Co-Al LDH/PoPD.

**SI Table:1 Electrochemical sensing of 4-NP, 2,4-DNP and UA using
Ag@Co-Al LDH/PoPD/GCE**

| **Samples** | **4-NP** | | | | **DNP** | | | | **UA** | | |
| --- | --- | --- | --- | --- | --- | --- | --- | --- | --- | --- | --- |
|  | I (µA) | | E (V) | | I (µA) | | E (V) | | I (µA) | E (V) | |
|  | I_pa_ | I_pc_ | E_pa_ | E_pc_ | I_pa_ | I_pc_ | E_pa_ | E_pc_ | I_pa_ | E_pa_ |  |
| Ag@Co-Al/PoPD/GCE  (**Absence of analyte**) | 10.18 | - | 0.17 | - | 11.61 | - | 0.14 | - | 2.56 | 0.28 |  |
| Ag@Co-Al/PoPD/GCE  (**Presence of analyte**) | 15.85 | -8.42  -36.91 | 0.23 | 0.08  -0.81 | 26.09  27.40  22.21 | -17.14  -44.84  -52.87 | 0.12  0.19  0.49 | -0.06  -0.49  -0.72 | 15.15 | 0.43 |  |

**Reference**

1. Dhansekaran, T., Padmanaban, A., Gnanamoorthy, G., Manigandan, R., Praveen Kumar, S., Stephen, A & Narayanan V. Recent Advances in polymer Supporting Layered Double Hydroxides nanocomposites for Electrochemical Biosensors, *Mater. Res. Express.* **5**, 014011 (2018).
2. Suresh, K., Vinoth K. R & Pugazhenthi, G. Processing and characterization of polystyrene nanocomposites based on Co-Al layered double hydroxide, *J. Sci. Adv. Mater. Dev,* **1**, 351-361 (2016).
3. Kannapiran, N., Muthusamy, A., Chitra, P., Anand, S & Jayaprakash, R. Poly(o-phenylenediamine)/NiCoFe_2_O_4_ nanocomposites: Synthesis, characterization, magnetic and dielectric properties, *J Mag Mag Mater,* **423,** 208-216 (2017).
